# Theoretical Design of Near-Infrared-Absorbing D-A-π-A Dyes with Modified Hagfeldt Donors: A DFT/TDDFT Study

**DOI:** 10.3390/ijms27177646

**Published:** 2026-08-26

**Authors:** Jing Huang, Zhixiang Hu

**Affiliations:** 1 Key Laboratory of Ecological Environment and Information Atlas (Fujian Provincial University), College of Environmental and Biological Engineering, Putian University, Putian 351100, China; 2 Fujian Provincial Key Laboratory of Ecological Impacts and Treatment Technologies for Emerging Contaminants, College, of Environmental and Biological Engineering, Putian University, Putian 351100, China; 3 Strait Institute of Flexible Electronic, Fujian Normal University, Fuzhou 350117, China

**Keywords:** dye-sensitized solar cells, near infrared, Hagfeldt donor, D-A-π-A configuration

## Abstract

Dye-sensitized solar cells based on Hagfeldt donor sensitizers achieve high open-circuit voltages through effective suppression of interfacial charge recombination. However, their absorption remains largely confined to the visible region, which limits further gains in the photocurrent and overall efficiency. This study addresses the issue of spectral limitation by designing six D-A-π-A organic dyes, **HJ101**~**HJ106**. These dyes are derived from the reference sensitizer **XY1** through systematic modification of the donor unit with anthracene and squaraine moieties combined with two benzothiadiazole-type auxiliary acceptors. Geometric structures, frontier molecular orbitals, absorption spectra, and excited-state charge transfer characteristics were investigated using density functional theory and time-dependent density functional theory. The results demonstrate that donor and acceptor modifications act synergistically to control the spectral direction and magnitude, with several dyes achieving pronounced redshifts extending into the near-infrared region while retaining thermodynamically favorable electron injection and regeneration driving forces. Among the designed structures, **HJ106**, which combines a squaraine-modified donor with a redshifting acceptor, exhibits the largest bathochromic shift and an extended excited-state lifetime. **HJ105** delivers the highest molar extinction coefficient and light-harvesting efficiency. Collectively, these findings identify squaraine-based donor engineering as the most promising strategy for near-infrared-responsive Hagfeldt-type sensitizers. These findings offer practical structural guidance for the development of next-generation dye-sensitized solar cell sensitizers with broadened spectral coverage.

## 1. Introduction

The power conversion efficiency of dye-sensitized solar cells (DSSCs) is determined by three factors: the light-harvesting range, the charge-separation efficiency, and the suppression of interfacial recombination [1,2,3]. The optimization of these three factors concurrently poses a significant challenge: donor architectures that broaden the absorption spectrum frequently demonstrate suboptimal performance in the suppression of recombination, whereas donors that exhibit strong recombination-blocking capability tend to confine absorption to the visible region. This trade-off remains a central, unresolved issue in sensitizer design, and progress on any one front rarely translates automatically into an improvement in overall device efficiency. This is because gains in the photocurrent are frequently offset by losses in the open-circuit voltage (*V_oc_*) or long-term stability [1,2,3].

The Hagfeldt donor, constructed on a tetraalkoxy-substituted triarylamine framework, effectively suppresses interfacial charge recombination [4]. This results in Hagfeldt-based sensitizers, such as **XY1**/**Y123**, **SM315**, and **LEG4**/**ADEKA-1**, exhibiting notably high open-circuit voltages and long-term stability [5,6,7,8,9]. The bulky alkoxy chains projecting from the triarylamine core create a steric shell around the anchored dye layer, physically retarding the approach of the redox electrolyte to the injected electrons and thereby extending the electron lifetime well beyond what is achievable with conventional triphenylamine donors [4]. For instance, **XY1** delivers a *V_oc_* of 1050 mV and a power conversion efficiency of 13.1% [5,6], whereas related architectures employing porphyrin and silyl-anchor collaborative sensitization strategies have pushed device efficiencies above 13% through similar recombination-suppression principles [7,8,9]. However, the absorption maximum of these Hagfeldt-donor dyes typically lies at approximately 500–550 nm, leaving the sensitizer largely unresponsive to near-infrared (NIR) photons, which carry a substantial fraction of solar energy. Consequently, the short-circuit current density and overall efficiency remain constrained by the narrow spectral window rather than by charge recombination losses. The Hagfeldt donor, however, successfully resolves the recombination problem without addressing narrow spectral coverage, which constitutes the first gap in this work.

**Figure 1:**
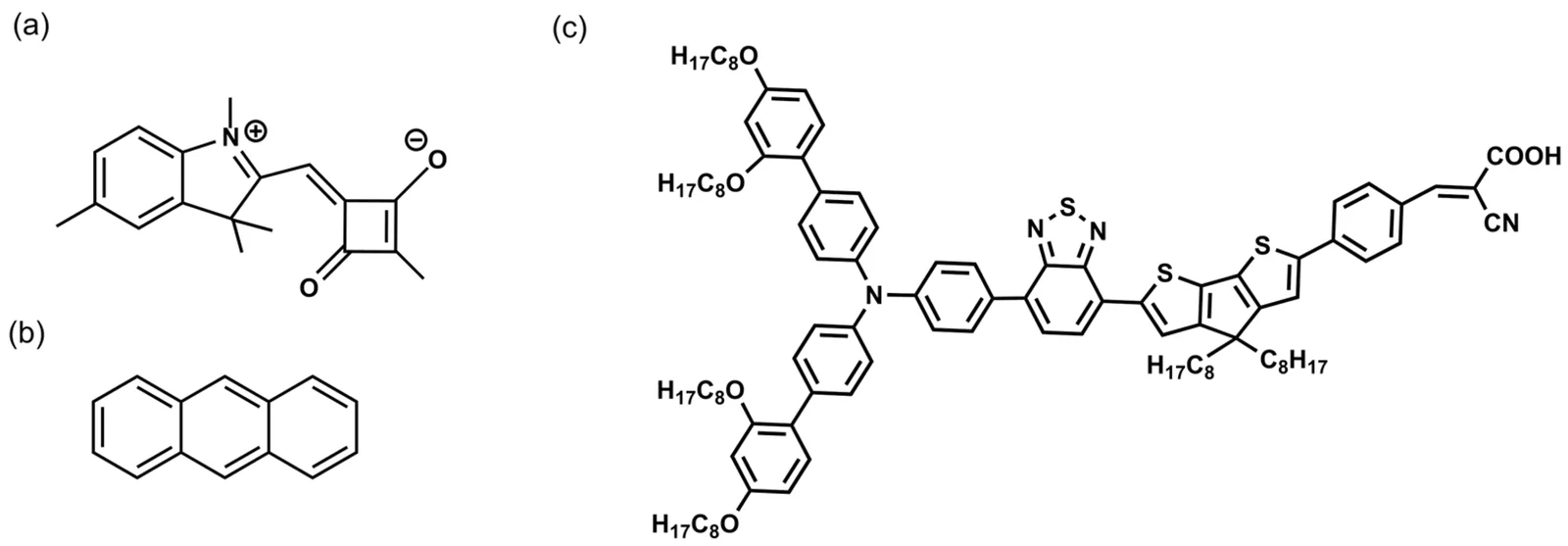
Chemical structures of the (**a**) squaraine core, (**b**) anthracene units and (**c**) dye **XY1**.

Squaraine cores and anthracene units present two structurally distinct pathways for extending NIR absorption. Each pathway has a substantial and growing body of supporting literature [10,11,12,13,14,15,16,17]. The squaraine chromophore (see Figure 1a) consists of a four-membered oxocyclobutenylium core that is flanked by electron-rich donor moieties. This structural feature leads to the occurrence of pronounced intramolecular charge transfer and molar extinction coefficients that frequently exceed 10 to the fifth per million per centimeter. These values are considerably higher than those typical of conventional triarylamine dyes [11,12]. The central squaraine ring functions as a robust internal acceptor. Consequently, the electronic properties and conjugation length of the flanking donor groups can be tuned, enabling precise regulation of the absorption edge. By incorporating benzoindole and related fused heterocyclic moieties, extended or unsymmetrical squaraine architectures have been demonstrated to extend the absorption spectrum from the visible range to 750–900 nm [10,13]. Systematic structure–property studies have further demonstrated that the position of alkyl substituents, the selection of anchoring groups, and the degree of donor asymmetry within the squaraine framework can each independently modulate both the absorption maximum and the device power conversion efficiency, thereby underscoring the high structural tunability of this chromophore class relative to conventional D-π-A dyes [12,14]. These properties have led to a resurgence of interest in squaraine dyes, not only in photovoltaics but also in bioimaging and photodynamic applications, where their strong and tunable NIR absorption is similarly exploited [10].

As illustrated in Figure 1b, anthracene employs a mechanistically distinct approach to NIR extension. Its rigid, planar polycyclic aromatic backbone extends π-conjugation along the donor-bridge axis and enhances intramolecular charge transfer, resulting in a markedly greater difference in dipole moment between ground and excited states when paired with a cyanoacrylic acceptor [15]. As anthracene does not possess a pronounced electron-withdrawing character, its spectral effect is typically more moderate than that of squaraine. However, it is more readily incorporated into conventional donor–bridge–acceptor frameworks without compromising the overall push—pull architecture. Furthermore, anthracene-based conjugated polymers and small-molecule dyes exhibit tunable bandgaps and redshifted absorption through the modulation of the substituent pattern and degree of ring fusion [15]. As demonstrated in previous studies, the use of related quinoxaline-fused porphyrin sensitizers has shown that extending planar aromatic conjugation near the donor or bridge region can significantly shift absorption into the near-infrared range while retaining favorable injection energetics [16]. The direct combination of anthracene with a modified Hagfeldt donor has already proven to be a viable experimental approach. A double-anthracene sensitizer built on a modified Hagfeldt donor has been shown to achieve a power conversion efficiency that exceeds 10% under copper-based redox mediation. This outcome confirms that the steric bulk of the fused ring system can be accommodated within the Hagfeldt donor scaffold without compromising its recombination-suppressing function [17].

Consequently, both structural units demonstrate evident potential for synergy with the Hagfeldt donor. However, it should be noted that the current study has two significant limitations. First, the majority of investigations examine only one structural motif in isolation, either squaraine or anthracene [18,19,20], without a direct, systematic comparison of the two strategies within the same donor scaffold and the same computational framework. This methodological shortcoming makes it difficult to judge which approach offers a more favorable balance between spectral extension and structural or electronic penalty. Second, theoretical treatment of the structure—property relationship at the density functional level remains limited for both chromophore families. Typically, the auxiliary acceptor unit is held fixed in existing designs, leaving the coupling between donor modification and acceptor strength largely unexamined.

**Figure 2:**
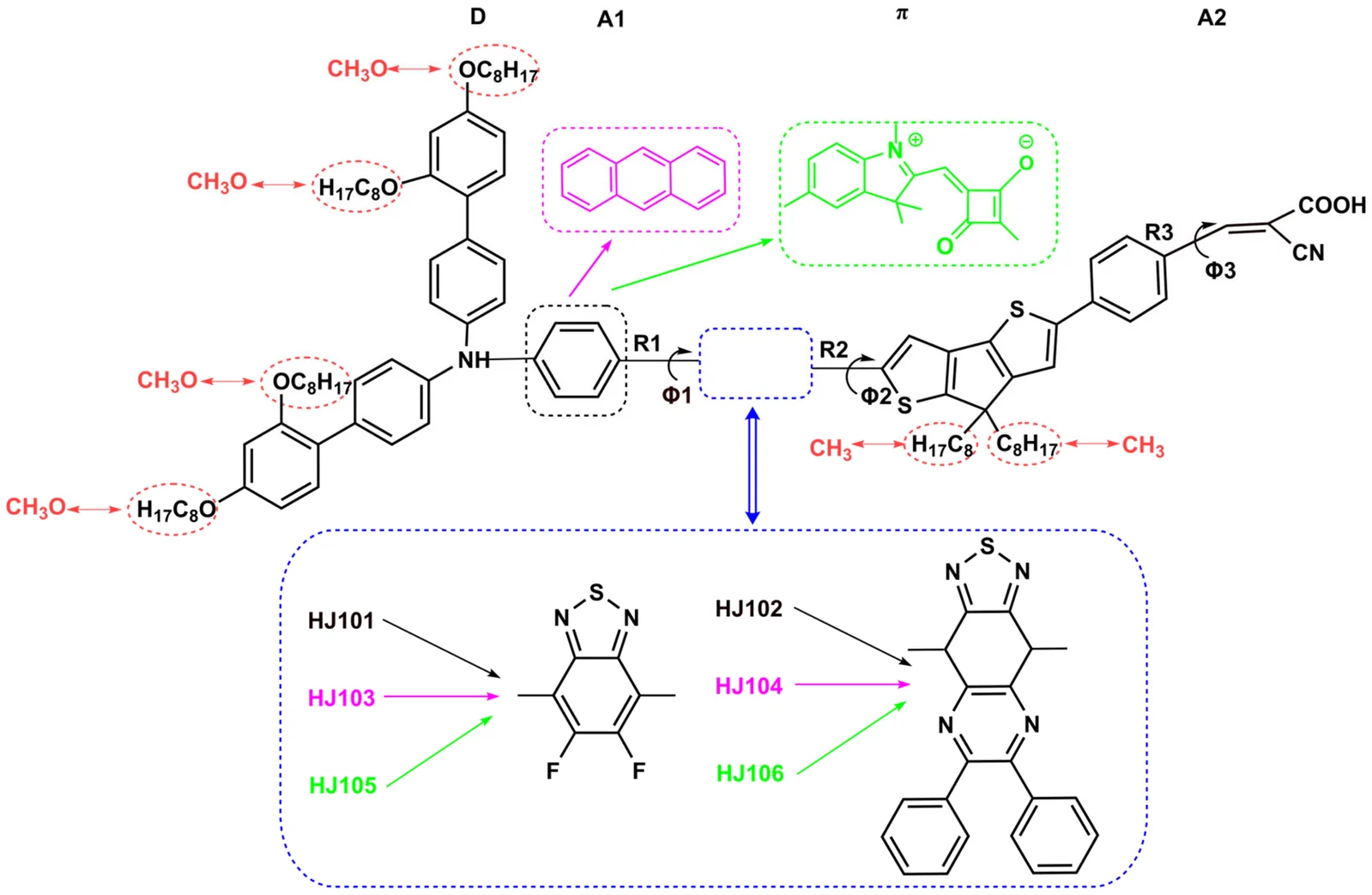
Schematic diagrams of the molecular structures of D-A-π-A-type organic photosensitizers (**HJ101**~**HJ106**). **R1** denotes the bond length (Å) connecting the donor (D) motif and the acceptor (A1) unit; **Φ1** denotes the corresponding dihedral angle between the donor (D) motif and the A1 unit. **R2** denotes the bond length (Å) connecting the A1 unit and the π-bridge; **Φ2** denotes the corresponding dihedral angle between the A1 unit and the π-bridge. **R3** denotes the bond length (Å) connecting the π-bridge and the acceptor A2 unit; **Φ3** denotes the corresponding dihedral angle between the π-bridge and the A2 unit. The numerical value of a dihedral angle is contingent upon the rotational sense in which the four defining atoms are traversed. A given torsion can be expressed as an acute angle θ or its obtuse supplement (180° − θ). All dihedral angles are uniformly reported in the acute range (0°~90°).

The novelty of the present study lies in preserving the recombination-suppressing advantage of the Hagfeldt donor while starting from the **XY1** scaffold shown in Figure 1c, replacing the phenyl unit within the donor with either a squaraine derivative or an anthracene moiety, and additionally incorporating two benzothiadiazole auxiliary acceptors of differing electronic strength. This process results in the generation of three distinct donor variants: a phenyl reference, an anthracene-modified donor, and a squaraine-modified donor. Each of these donor variants is then paired with both acceptors, resulting in a total of six D-A-π-A dyes, designated **HJ101**~**HJ106**. The design’s integration of donor modification and acceptor variation within a unified computational framework enables the evaluation of these two NIR extension strategies under identical conditions. This approach elucidates their respective effects on the frontier orbital distribution, energy-level alignment, spectral redshift, and electron-injection driving force. Furthermore, it determines whether donor modification and acceptor strength act synergistically or antagonistically. The objective of this study is to utilize density functional theory (DFT) and time-dependent density functional theory (TDDFT) to establish structural criteria for sensitizers that exhibit a high open-circuit voltage in conjunction with NIR responsiveness. This approach directly addresses the fundamental question of which extension strategy, in conjunction with which acceptor strength, optimally balances the light-harvesting range against the charge-separation performance without compromising the recombination-suppressing benefit of the Hagfeldt donor. Since the cyanoacrylic acid anchor is identical across all six dyes and its well-established stable bidentate bridging adsorption on the semiconductor surface [21,22,23], the present calculations focus on isolated dye molecules, where the relative photovoltaic trends are expected to be preserved upon adsorption [24,25]. To minimize computational expense, all C_8_H_17_ groups on the donor were substituted with CH_3_O, and the C_8_H_17_ substituents in the π-bridge were replaced with CH_3_ throughout the DFT/TDDFT calculations, as illustrated in Figure 2.

## 2. Results and Discussion

### 2.1. Geometric Configuration

Table 1 provides a concise overview of the key bond lengths and dihedral angles of the optimized dye molecules. The spatial configuration of the donor, π-bridge, and acceptor units profoundly influences the orbital overlap and conjugation efficiency. Consequently, dihedral angle analysis offers preliminary insight into the molecular framework alterations resulting from anthracene substitution and squaraine substitution at the donor site relative to the **XY1** dye and its benzene-donor analogs, **HJ101** and **HJ102**.

The **XY1** structure displays values of 34.2°, 9.9°, and 0.4°, which are consistent with a partially planar D-π-A backbone. The introduction of the 5,6-difluoro-2,1,3-benzothiadiazole (DFBTD) acceptor in **HJ101** results in an increase in Φ1 to 42.3°, accompanied by a significant reduction in Φ2 to 0.3°. This observation suggests that the auxiliary acceptor enhances planarity in the vicinity of the π-bridge while concurrently inducing a slight twisting of the donor–acceptor junction. Subsequent to the replacement of the donor phenyl ring with an anthracene unit, the D–A1 dihedral angle, designated as Φ1, underwent a substantial increase, from 34.2° in **XY1** to 79.8° in **HJ103**, representing an increase of 45.6°. This outcome is attributed to the increased steric volume of the fused anthracene ring, which introduces intramolecular hindrance at the donor–acceptor interface and disrupts local planarity. A similar but more pronounced trend is evident in HJ104, where the parameter of interest, denoted by Φ1, reaches a value of 85.3°, and the analogous parameter, designated as Φ2, increases to 26.3°, representing the maximum observed value among the six dyes under consideration. This behavior is in contrast to that observed in previous computational studies of anthracene-based D-π-A dyes, wherein fused thienoacene or cyclopentadithiophene spacers were utilized to extend conjugation while preserving planarity and achieving a device efficiency as high as 12.6% [26]. The absence of a buffering spacer in **HJ103** and **HJ104** appears to be the primary source of the torsional penalty observed here.

In contrast, the values of the squaraine-modified dyes **HJ105** and **HJ106** are significantly lower, at 35.8°and 42.2°, respectively, and approach those of the benzene-donor reference. This observation persists despite the structural bulk of the squaraine unit. In contrast to the linearly fused anthracene ring, the four-membered squaraine core does not introduce comparable steric strain at the donor–acceptor junction. This allows the donor and the π-bridge to remain nearly coplanar. Among all the designed dyes, **HJ105** demonstrates the most regular planarity. This geometric outcome supports one of the central claims raised in the introduction, namely, that squaraine substitution offers a structurally gentler route to spectral extension than direct anthracene fusion does. This is because it avoids the steric penalty associated with rigid polycyclic donor extension while still enabling strong intramolecular charge transfer.

### 2.2. Frontier Molecular Orbital Analysis and Excited-State Configurations

**Figure 3:**
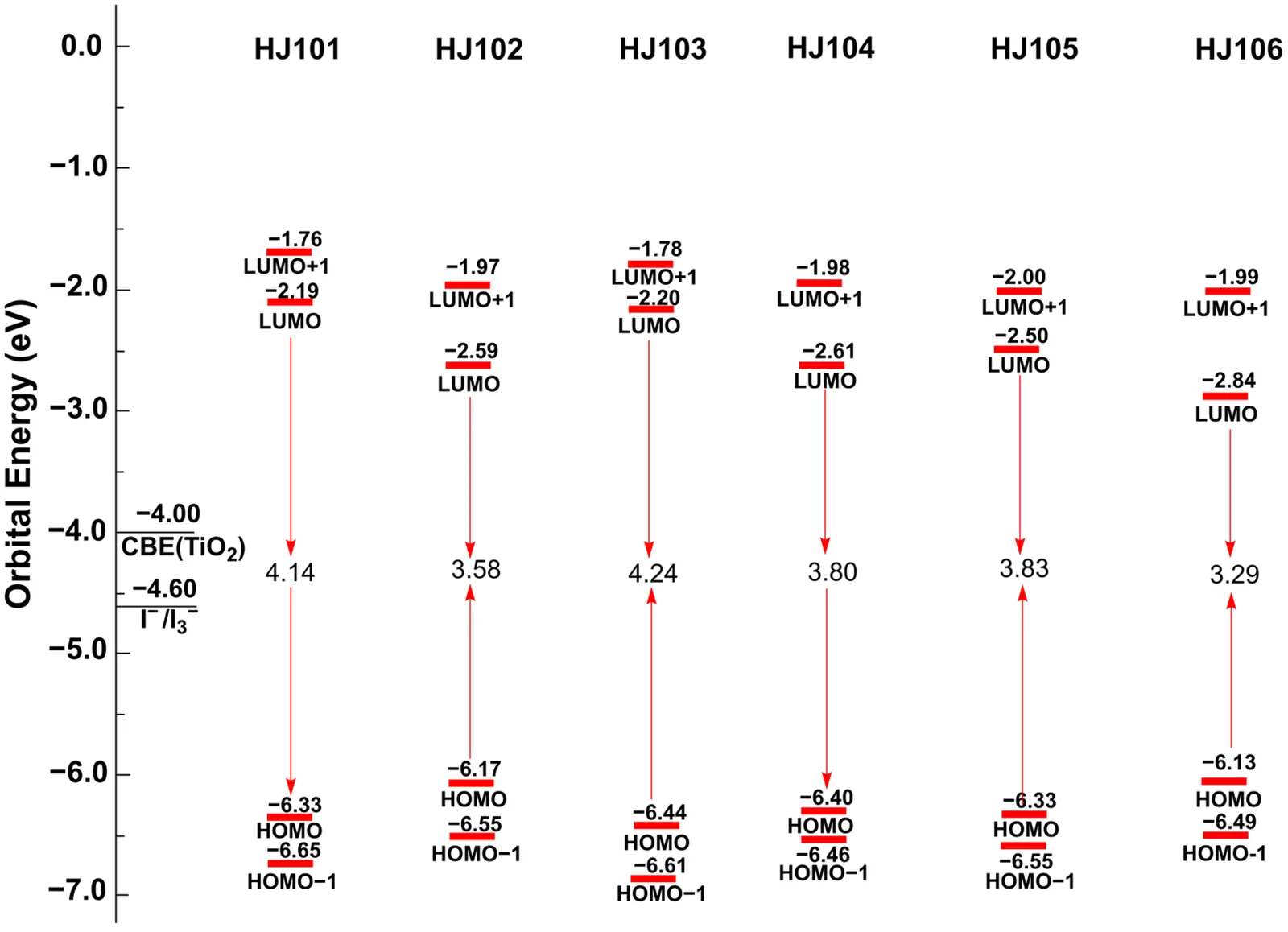
Schematic diagram of the front-line molecular orbital energy levels of an organic dye molecule.

As illustrated in Figure 3, which depicts the front-line molecular orbital energy levels of the dyes, the HOMO–LUMO energy gap, denoted as ∆*E_g_*, is demonstrated. A comparison of the energy level compatibility of dye molecules, titanium dioxide semiconductors, and redox electrolytes provides a foundation for assessing the suitability of dyes for use in DSSCs. As illustrated in Figure 3, the LUMO energy levels of all the dyes exceed the conduction band energy level of titanium dioxide (−4.0 eV), while their HOMO energy levels are lower than the I^−^/I_3_^−^ electrochemical potential (−4.6 eV) [27]. This process ensures that the dyes can transfer electrons to titanium dioxide, allowing the oxidized dyes to undergo reduction.

Table 2 presents a comprehensive list of the vertical excitation energies (∆*E_exc_*) and their corresponding dominant orbital configurations. TDDFT calculation results indicate that these excited states generally exhibit multiconfigurational electronic structure characteristics. The S_1_ state of the **HJ106** dye is attributed primarily to the HOMO→LUMO transition, which accounts for more than 87.9% of the contribution. In contrast, the other dyes exhibit distinct orbital mixing transitions, such as H−1→L and H→L+1. Notably, orbital mixing does not inherently diminish the charge transfer (CT) efficiency. In fact, if the pertinent orbitals (e.g., HOMO−1 or LUMO+1) retain sufficient spatial overlap between the donor and acceptor units, these excited states can effectively facilitate the intramolecular charge transfer (ICT) process.

### 2.3. Absorption Spectra and Excited-State Parameters

**Figure 4:**
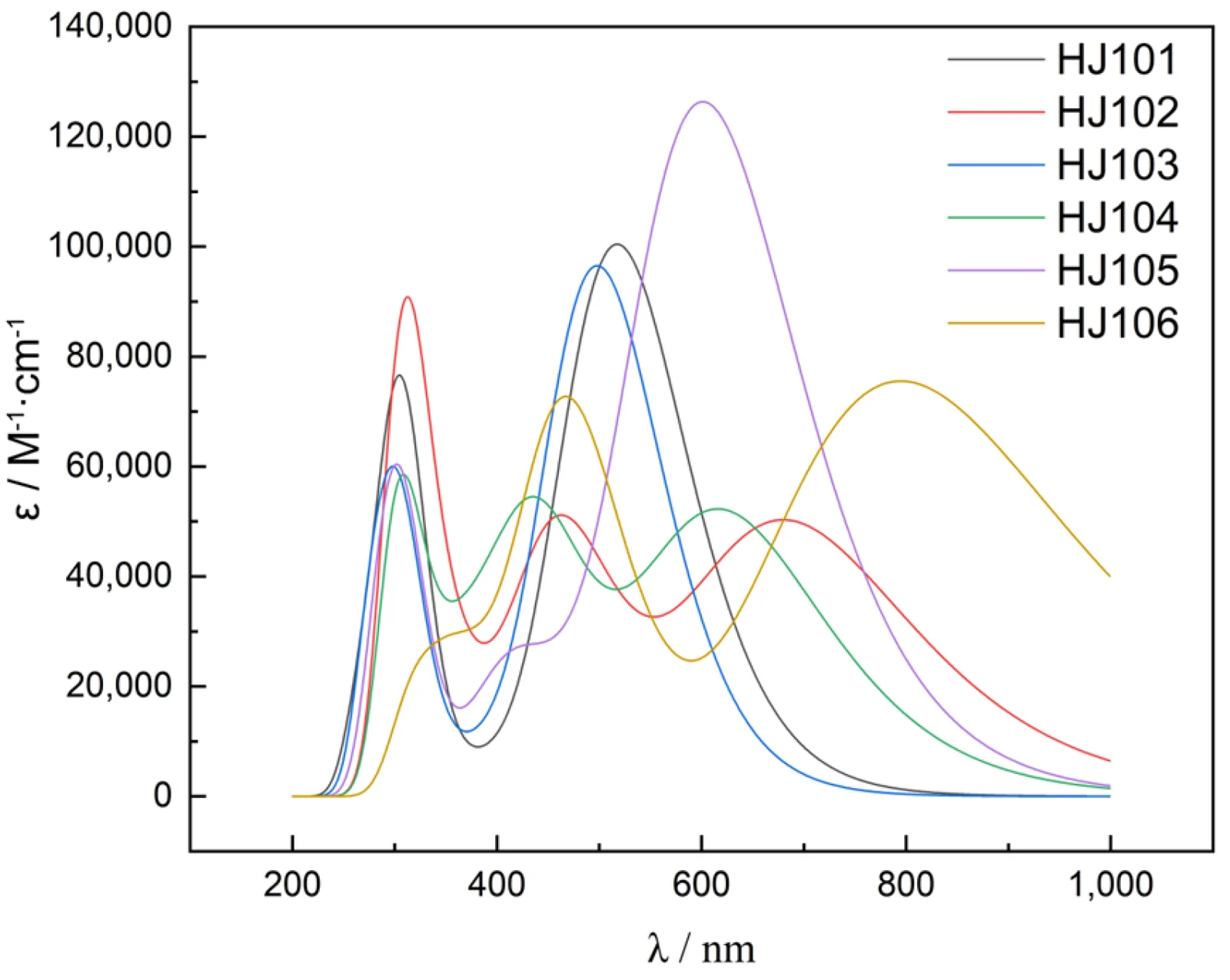
UV—Vis absorption spectra of **HJ101**–**HJ106**.

In terms of the physical optical properties and absorption spectra, the simulated absorption parameters (including the maximum absorption wavelength *λ_max_*, the molar extinction coefficient *ε*, the light-harvesting efficiency *LHE*, and the excited-state lifetime *τ*) are summarized in Table 3, and the corresponding UV—visible absorption spectra are shown in Figure 4. In this study, *λ_max_* is defined as the excitation wavelength corresponding to the electronic transition with the maximum oscillator strength in the visible or near-infrared region. As shown in Table 2, the S_1_ excited state is the primary contributor to all the dye molecules studied.

Two opposite spectral trends emerge depending on the identity of the auxiliary acceptor. Compared with **XY1**, **HJ101** and **HJ103**, which carry the DFBTD acceptor, exhibit blueshifts of 16 nm and 36 nm, respectively. This shift can be attributed to the electron-withdrawing character of the DFBTD unit, which reduces the effective conjugation length. In contrast, the absorption maxima of the second BTD derivative acceptors, **HJ102**, **HJ104**, **HJ105**, and **HJ106**, redshift from 67 nm to 261 nm, thereby extending beyond 600 nm, and, for **HJ106**, to 795 nm, thus positioning it within the near-infrared region.

These findings directly address the design question raised in the introduction, namely, whether donor engineering and acceptor engineering act synergistically within the same Hagfeldt-based scaffold. The data demonstrate that acceptor identity is the predominant switch regulating the direction of the spectral shift, whereas donor identity modulates the magnitude and extinction coefficient once a redshifting acceptor is present. Among the redshifted dyes, **HJ106** exhibited the most significant bathochromic shift of the series. This phenomenon is similar, albeit on a more modest scale, to reports on extended polymethine squaraine sensitizers. In these cases, stepwise thiophene insertion between the donor and acceptor shifts the charge transfer band from 541 nm to 833 nm, with extinction coefficients that exceed 1 × 10^5^ M^−1^·cm^−1^ [26]. The present results indicate that comparable near-infrared extension can already be achieved by pairing a squaraine-modified Hagfeldt donor with a matched auxiliary acceptor, without additional π-extension units, representing a more synthetically economical strategy.

The anthracene-modified dyes, on the other hand, exhibit a distinct pattern. **HJ104** redshifts by 82 nm relative to that of **XY1**, reaching 616 nm, while its extinction coefficient decreases to 5.23 × 10^4^ M^−1^·cm^−1^, which is the lowest among the six dyes and approximately half that of HJ105. These findings align with the geometric distortion previously observed, wherein steric hindrance at the anthracene donor junction leads to a reduction in planarity and orbital overlap along the charge transfer axis. Reports on fused-ring anthracene DSSC sensitizers have demonstrated that comparable redshifts can be obtained through thienoacene fusion at the donor with reported device efficiencies up to 12.6%, but only when the fused unit is inserted through a spacer that preserves planarity [13]. As indicated by related studies on hydroxyl-functionalized anthracene D-π-A dyes, spectral redshifts and light-harvesting gains are contingent on the manner in which substitution affects the HOMO energy and intramolecular charge transfer characteristics rather than on the extension of conjugation alone [10]. This comparison suggests that the direct fusion strategy adopted for **HJ103** and **HJ104** captures part of the spectral benefit of anthracene conjugation while sacrificing some of the extinction coefficient gain that a better-planarized anthracene linkage might otherwise provide.

**HJ105** exhibits the highest molar extinction coefficient in the series, 12.64 × 10^4^ M^−1^·cm^−1^, accompanied by a light-harvesting efficiency of 99.9%, signifying that the squaraine donor, when conjugated with the DFBTD acceptor, facilitates the most efficient light capture per absorbed photon among all the designed structures, despite its less redshifted absorption maximum at 601 nm than **HJ106** does. The excited state lifetimes range from 1.58 ns to 5.67 ns, with **HJ102** and **HJ106** exhibiting the longest values, both exceeding 5 ns. This is advantageous for sustaining the charge-separated state prior to electron injection.

When considered collectively, squaraine substitution provides two distinct advantages over anthracene substitution within the same Hagfeldt-donor D-A-π-A framework. It has been demonstrated to preserve molecular planarity with greater efficacy and to achieve a more favorable balance between the magnitude of redshift and the extinction coefficient. In contrast, anthracene substitution primarily achieves a redshift at the cost of oscillator strength. This provides a direct, structure-based answer to the comparative question posed in the introduction regarding which near-infrared extension strategy performs better under matched donor and acceptor conditions.

### 2.4. Molecular Orbital Analysis

**Figure 5:**
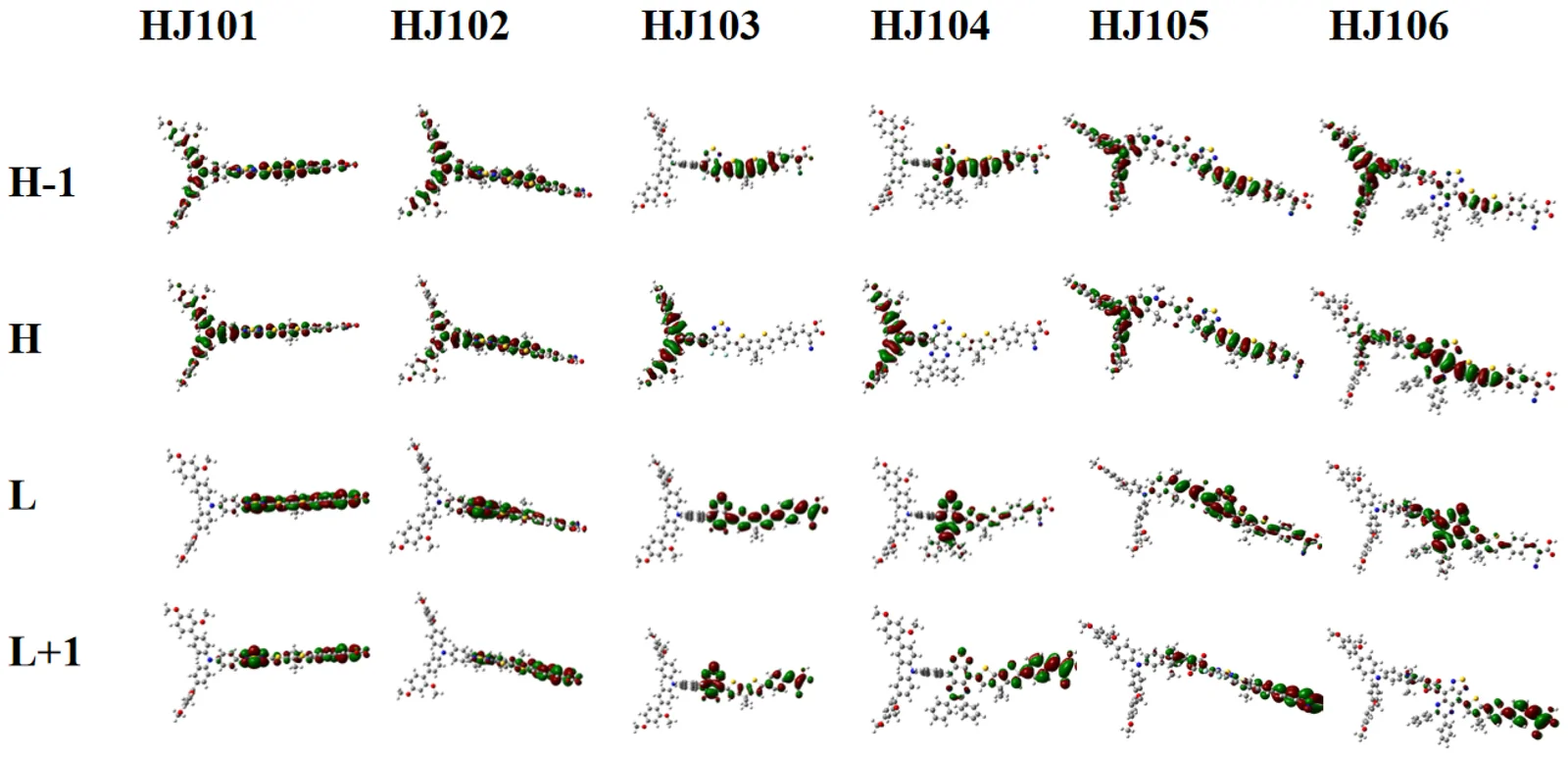
Front-of-band orbitals of the **HJ101**–**HJ106** dye molecules. The green and red colors represent the positive and negative phases of the wavefunctions, respectively.

As shown in Figure 5, in the series of D-A-π-A-type dyes **HJ101**–**HJ106**, the HOMO electron cloud is primarily distributed in the donor D unit and the π-bridge region, whereas the LUMO electron cloud exhibits a marked delocalized expansion toward the electron acceptor A region at the molecular terminus. This orbital distribution characteristic is consistent with the orbital patterns of the dye systems studied in this project. As demonstrated in Table 2, within this category of dye molecules, the transition of electrons from the HOMO to the LUMO constitutes the predominant mechanism for light energy absorption [28].

As demonstrated in Figure 5, in the designed D-A-π-A-type molecules, the front-line molecular orbitals exhibit substantial spatial polarization: the highest occupied molecular orbital (HOMO) is predominantly localized in the electron-rich donor unit, the adjacent acceptor (A1), and the π-conjugated bridge moiety, whereas the lowest unoccupied molecular orbital (LUMO) is concentrated in the A1, π-bridge, and terminal acceptor A2 regions. This asymmetric electron density distribution is consistent with the characteristics of light-induced, directed intramolecular charge migration and represents a hallmark electronic structural feature of typical, highly efficient push—pull chromophores. By focusing on the aforementioned reference molecule, this illustration effectively reveals the regulatory mechanisms by which structural modifications to the auxiliary and terminal acceptors control the electron density distribution and spectral behavior while avoiding the redundancy associated with presenting a large number of similar derivatives with comparable orbital distribution patterns. To ensure the comprehensiveness and traceability of the study, front-row orbital diagrams for the remaining sensitizers have been incorporated into the Supplementary Information.

In the context of solar cells, the photoelectric properties of the dye are pivotal in determining the overall performance of the cell. When light strikes the dye, the donor portion of the dye molecule transfers electrons to the acceptor portion, causing positive and negative charges to separate. The separated electrons then enter the semiconductor film and ultimately flow toward the cell’s external circuit, thereby generating an electric current. The ability of electrons to enter the semiconductor conduction band can be measured by the injection driving force (∆*G_inj_*), whereas the regeneration of the dye after electron loss depends on the regeneration driving force (∆*G_reg_*). As demonstrated in Table 4, for the six dyes **HJ101**–**HJ106**, the minimum values of ∆*G_inj_* and ∆*G_reg_* are −1.16 eV and −1.50 eV, respectively. According to Islam’s theory [29], effective electron injection and regeneration are contingent upon the conditions of ∆*G_inj_* being less than −0.8 eV and ∆*G_reg_* being less than −0.5 eV. A comprehensive analysis of the thermodynamic parameters revealed that the energy level arrangement of this series of dyes is in full accordance with the operational requirements for high-efficiency dye-sensitized solar cells. First, the excited-state energy level of the dyes exceeds the bottom of the semiconductor conduction band, thereby ensuring a sufficient driving force for electron injection. Second, the ground-state oxidation potential of the dyes is lower than that of the electrolyte redox pair, thus facilitating rapid regeneration of the dye cations. The combination of these two thermodynamic advantages establishes an energetic foundation for each dye to achieve highly efficient photoelectric conversion.

### 2.5. Transition Density Matrix (TDM) Analysis

**Figure 6:**
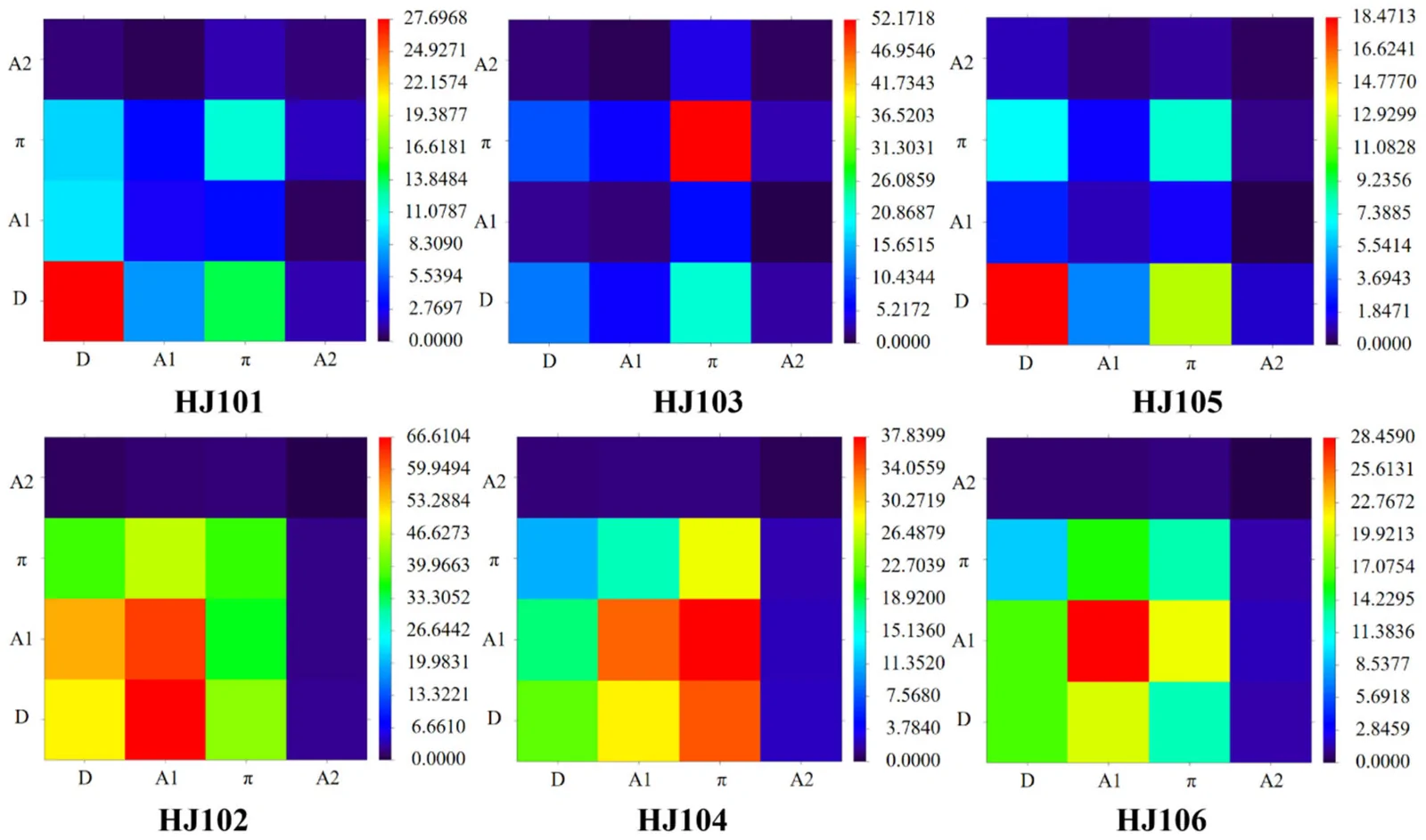
Heatmap of the fragment transition density matrix (TDM), illustrating the charge transfer characteristics of key photosensitizers.

To resolve how the donor structure reorganizes the excited-state charge transfer pathway beyond what is visible in static orbital plots, the S_0_ to S_1_ transition density matrices were computed for the three donor pairs: benzene (**HJ101** and **HJ102)**, anthracene (**HJ103** and **HJ104**), and squaraine (**HJ105** and **HJ106**), as shown in Figure 6. These were then decomposed into donor, first acceptor, π-bridge, and second acceptor fragments.

The benzene donor pair demonstrates robust diagonal elements, accompanied by significant off-diagonal coupling across multiple pathways, including donor-to-bridge, donor-to-first-acceptor, and bridge-to-bridge transfer. This pattern reflects a locally dominated excited state that nonetheless retains efficient cross-fragment communication, which is favorable for maintaining fast charge separation while limiting recombination losses.

Compared with the benzene pair, the anthracene donor pair significantly increased both the diagonal and off-diagonal intensities, indicating enhanced delocalization from the extended aromatic donor. The key donor-to-bridge and bridge-to-bridge pathways maintain their strength rather than being suppressed by the added delocalization, indicating that the anthracene donor enriches the coupling network rather than replacing local excitation with diffuse, weakly coupled character. This multipath enhancement is consistent with the broadened and redshifted absorption observed for **HJ103** and **HJ104**, and it offers a mechanistic explanation for why anthracene substitution, despite its geometric distortion, still contributes positively to the spectral response. A comparable coupling enhancement upon anthracene extension was reported for Hagfeldt-donor sensitizers bearing double anthracene units, which achieved power conversion efficiencies above 10% once molecular aggregation was suppressed through bulky donor modification [30]. These findings underscore that the steric drawbacks identified in the present series can, in principle, be mitigated through further donor engineering.

The squaraine donor pair demonstrates the most extensive spatial delocalization of the three groups, with both diagonal and off-diagonal elements dispersed across a wider set of fragment pairs. However, compared with the benzene and anthracene systems, this pair has a reduced overall transfer intensity. This phenomenon is indicative of a more globally distributed excited state, where no individual transition channel predominates. This is expected to prolong the excited-state lifetime and mitigate the probability of rapid interfacial recombination, a hypothesis that is consistent with the comparatively longer lifetime of **HJ106**. These findings align with observations on indenoquinaldine-based unsymmetrical squaraine sensitizers, where extended donor delocalization has been associated with a broadened near-infrared response and stable excited-state behavior in device measurements [31,32]. This may also imply a somewhat lower peak injection driving force relative to a fully localized transition, a trade-off that is relevant when selecting a donor strategy for a specific device configuration.

In essence, the three donor strategies that have been examined herein correspond to discrete structure–property regimes, as opposed to incremental variations in a singular design principle. The benzene donor exhibits a propensity for local excitation, accompanied by efficient multipath injection. In contrast, the anthracene donor demonstrates a preference for enhanced delocalization, resulting in a broadened spectral response, albeit at the expense of planarity and the extinction coefficient. The squaraine donor, on the other hand, favors wide-area delocalization, characterized by a high extinction coefficient, robust planarity retention, and an extended excited-state lifetime. However, this behavior may incur a cost in terms of injection intensity within a singular dominant channel. This three-way comparison, performed under an identical Hagfeldt donor scaffold and an identical pair of auxiliary BTD acceptors, provides direct structural evidence for the design question raised in the introduction. It also demonstrates that the preferred strategy depends on which photovoltaic parameter, spectral range, extinction coefficient, or excited-state lifetime is prioritized for the target device.

### 2.6. Synthetic Feasibility of the Promising Candidates

**HJ106**, which combines a squaraine-modified donor with a redshifting acceptor, exhibits the largest bathochromic shift and an extended excited-state lifetime, while **HJ105** delivers the highest molar extinction coefficient and light-harvesting efficiency. Collectively, these two dyes represent the most promising candidates identified in this work. Despite the purely theoretical nature of the present study, it is worthwhile to briefly consider the feasibility of synthesizing **HJ105** and **HJ106**, along with the anthracene-modified dyes **HJ103** and **HJ104**, using methodologies previously reported [33,34]. Sensitizers bearing a bulky Hagfeldt-type triarylamine donor combined with a squaraine core have in fact already been prepared experimentally. Nawghare and coworkers recently reported the synthesis of an unsymmetrical squaraine dye carrying a Hagfeldt-type donor. This dye was obtained through palladium-catalyzed Buchwald–Hartwig amination of a brominated indolium precursor with a bis-biphenyl diarylamine. The resulting Fischer base was then condensed with a carboxylic acid-functionalized semisquaric acid under Dean–Stark conditions. This process affords the target dye in a workable, albeit moderate, yield [33]. This route utilizes a similar class of building blocks as **HJ105** and **HJ106**, namely an indoline-derived Fischer base bearing the Hagfeldt donor and a semisquaric acid unit with the auxiliary acceptor. This finding suggests that squaraine-modified Hagfeldt-donor dyes of this general type are synthetically feasible rather than purely hypothetical. Separately, anthracene-modified Hagfeldt-donor dyes, which bear a strong resemblance to **HJ103** and **HJ104**, were also meticulously synthesized and integrated into functional devices. These devices exhibited power conversion efficiencies that surpassed eight percent. This remarkable feat was achieved through a sophisticated process involving direct arylation and cross-coupling of an anthracene-ethynyl bridge onto the alkoxylated triarylamine donor. This outcome serves to reinforce the general compatibility of alkoxy-substituted Hagfeldt-donor scaffolds, whether they are anthracene- or squaraine-modified, with the synthetic transformations that are pertinent to the current series [34]. It is important to note that **HJ105** and **HJ106** also necessitate the installation of a benzothiadiazole-type auxiliary acceptor between the squaraine core and the terminal cyanoacrylic acid group. This step is not present in the reported squaraine synthesis. In addition, **HJ103** and **HJ104** would similarly require the coupling of this same auxiliary acceptor onto the anthracene-ethynyl bridge. In both cases, the steric bulk of the donor and the reduced planarity noted for the anthracene-modified structures may necessitate further optimization of coupling conditions and reaction temperature to maintain acceptable yields. It is evident that, while **HJ103** through **HJ106** have not yet been prepared, each of the individual synthetic operations they would require has already been demonstrated separately in closely related systems. These operations include donor arylation, Fischer base formation, anthracene-bridge coupling, semisquaric acid condensation, and auxiliary acceptor installation. This suggests that all four candidates should be accessible using currently available synthetic methodology.

## 3. Materials and Methods

### 3.1. Geometric Optimization

All calculations were performed using the Gaussian 16 program [34], with solvation effects fully accounted for throughout the calculations in CH_2_Cl_2_ with a dielectric constant of 9.08. Molecular structure optimization was executed by means of the IEFPCM continuum solvation model [35,36], which conceptualizes the solvent as a polarizable dielectric continuum. This model facilitates the incorporation of the electrostatic response of the surrounding medium into the electronic structure calculation in a self-consistent manner. In light of the discrepancies in precision among disparate DFT functionals in the context of donor–acceptor charge transfer systems, the hybrid PBE0 functional [37], in conjunction with the 6-311+G** basis set, was identified as the optimal approach for the optimization of the ground-state geometry of all seven dye molecules, **XY1** and **HJ101**~**HJ106**. This combination of functional and basis sets has been previously validated in a series of computational studies of organic sensitizers, including the Hagfeldt-donor and D-A-π-A types. These studies demonstrated that PBE0/6-311+G**-optimized geometries were capable of reproducing experimental bond lengths, dihedral angles, and overall molecular planarity with a high degree of accuracy. This finding supports the continued utilization of this combination for the current series of donor-modified dyes [38,39,40,41,42,43]. Subsequently, frequency calculations were performed on all the optimized structures at the same level of theory. This was done to confirm that each conformation corresponds to a true minimum on the potential energy surface. The results of these calculations revealed that all the vibrational frequencies were positive. This verified the stability of the optimized geometries.

### 3.2. Excited-State Energy Calculations

TDDFT is widely applied for the calculation of molecular vertical excitation energies. TDDFT is reasonably accurate and efficient and is employed in the prediction of absorption spectra in organic photosensitizers. In this study, modified D-A-π-A dyes containing a Hagfeldt donor were selected as the target molecules for excited-state analysis. Subsequent to ground-state geometry optimization at the PBE0/6-311+G** level, single-point TDDFT excitation energy calculations were executed using the long-range corrected CAM-B3LYP functional [44] in conjunction with the 6-311+G** basis set. The CAM-B3LYP functional [44] was selected because of its incorporation of distance-dependent exchange correction, a feature that mitigates the self-interaction error frequently observed in conventional hybrid functionals within extended charge transfer systems. This characteristic renders it especially well suited to the intramolecular donor-to-acceptor excitations that are hallmarks of D-A-π-A dyes. The accuracy of the PBE0/6-311+G** geometry combined with the CAM-B3LYP/6-311+G** excited-state protocol has been established in prior work. In that work, the calculated absorption maxima for structurally related Hagfeldt-donor sensitizers were found to agree closely with the experimentally measured UV—vis spectra. This justifies the adoption of the PBE0/6-311+G** geometry combined with the CAM-B3LYP/6-311+G** excited-state protocol for the present series without further benchmarking [38,39,40,41,42,43]. All excited-state calculations were carried out in a CH_2_Cl_2_ solvent environment using the IEFPCM solvation model [35,36], which is consistent with the solvation treatment applied during geometry optimization, to maintain internal consistency between the ground-state and excited-state descriptions.

### 3.3. Performance Calculations

The ease of electron transfer can be determined by the energy difference between the HOMO and LUMO. The ease with which electrons transition is directly proportional to the energy difference between the levels; the lower the energy difference is, the easier it is for electrons to transition. Consequently, the lower the energy difference and the greater the difficulty of electron transition and transfer are, the more favorable the photoelectric conversion process. In DSSC systems, the photoelectric performance of the dye is ultimately evaluated using the device’s photoelectric conversion efficiency (*η*) as a comprehensive metric. The primary determining factors of this metric include a combination of parameters, such as short-circuit current density, open-circuit voltage, fill factor, and incident light intensity. The expression is as follows [27,45]:

Among these, the short-circuit current density (*J_sc_*) is a pivotal parameter affecting device efficiency. Its physical nature can be characterized by integrating the light capture and charge injection processes at different wavelengths, as expressed by [28,46]:

(1) $$\eta \;=\;\frac{\mathrm{FF}V_{\mathrm{oc}}J_{\mathrm{sc}}}{P_{\mathrm{inc}}}$$

In this equation, *Φ_inj_* denotes the electron extraction efficiency, *η_coll_* signifies the charge collection efficiency, and *LHE*(*λ*) represents the light-harvesting efficiency.

(2) $$J_{\mathrm{SC}}=\mathrm{LHE}\left(\lambda \right)\Phi_{\mathrm{inj}}\eta_{\mathrm{coll}}d\lambda$$

Among the numerous performance parameters, light-harvesting efficiency (*LHE*) and the lifetime of the first excited state (*τ*) are the key factors determining the performance of the dye in DSSCs. The *LHE* of the dye at its maximum absorption wavelength can be calculated using the oscillator strength at that wavelength, and the *LHE* can be determined using the following equation [47]:

(3) $$\mathrm{LHE}\;=\;1\;-\;10^{-f}$$

In this model, *f* denotes the oscillator strength of the dye at its maximum absorption peak, reflecting the probability of this electronic transition.

The injection driving force is evaluated by the change in Gibbs free energy (∆*G_inj_*), which is directly related to the electron injection efficiency (*Φ_inj_*). The expression of ∆*G_inj_* is given by [48]:

(4) $$∆G_{\mathrm{inj}}\;=\;E_{{\mathrm{dye}}^{*}}\;-\;E_{\mathrm{CB}}$$

In the equation, E_dye_* is representative of the potential of the dye in its excited state, and *E_CB_* is the conduction band energy level of the semiconductor. For the TiO_2_ semiconductor, the conduction band energy level has been experimentally measured to be −4.0 eV [49].

The regenerative capacity of the dye in the I^−^/I_3_^−^ electrolyte is governed by the ∆*G_reg_* reaction, which is expressed as follows [50]:

(5) $$∆G_{\mathrm{reg}}\;=\;E_{\mathrm{redox}}-{\;E}_{\mathrm{dye}}$$

Here, Eredox is the redox potential of the I^−^/I_3_^−^ electron pair, with an experimentally measured value of −4.7 eV [28].

The excited-state lifetime (*τ*) is another key factor affecting charge transfer efficiency. Generally, a longer excited-state lifetime is more conducive to the separation and transfer of photogenerated charges, thereby reducing recombination losses. The excited-state lifetime (*τ*) [28] can be calculated using the following formula:

(6) $$\tau \;=\;1.499/fE^{2}$$

In this equation, *f* represents the oscillator strength of the excited state under consideration, and *E* represents the perpendicular excitation energy (in cm^−1^).

## 4. Conclusions

By utilizing **XY1** as the reference framework, the present study designed and systematically evaluated six D-A-π-A organic dye sensitizers, **HJ101**–**HJ106**. The donor unit of these dye sensitizers was modified with benzene, anthracene, or squaraine moieties and paired with two structurally distinct benzothiadiazole auxiliary acceptors. All the designed dyes retain a partially planar donor-π-bridge-acceptor configuration. The DFBTD-containing dyes **HJ101**, **HJ103**, and **HJ105** exhibit the most favorable planarity, whereas the anthracene-modified dyes **HJ103** and **HJ104** display a pronounced increase in the donor–acceptor dihedral angle because of steric hindrance from the fused aromatic ring. The frontier orbital energy levels of all the dyes satisfy the thermodynamic requirements for efficient DSSC operation, with electron injection and regeneration driving forces well beyond the effective thresholds. These findings confirm that neither donor modification compromises the energetic viability of the sensitizers. Spectroscopically, dyes bearing the DFBTD acceptor undergo a moderate blueshift relative to **XY1**, whereas those bearing the second BTD derivative acceptor show substantial redshifts of up to 261 nm, extending absorption well into the near-infrared region. Transition density matrix analysis revealed that the three donor types establish distinct excited-state charge transfer networks, ranging from a locally concentrated but multipath-connected pattern for the benzene donor to an enhanced, delocalized coupling network for the anthracene donor and a widely dispersed, longer-lived excited state for the squaraine donor. Taken together, these structural and electronic trends indicate that **HJ106** is the most effective sensitizer for harvesting near-infrared light, exhibiting the greatest spectral redshift and an extended excited-state lifetime. In contrast, HJ105 is distinguished by its remarkably high extinction coefficient and near-unity light-harvesting efficiency. These two squaraine-based dyes represent the most promising candidates identified in this work. They demonstrated that squaraine donor engineering, when coupled with an appropriately matched auxiliary acceptor, offers an effective and synthetically accessible route to extend the spectral response of Hagfeldt-type sensitizers without sacrificing charge injection or regeneration performance. This study thus provides a systematic theoretical basis for donor engineering in D-A-π-A dye sensitizers and identifies squaraine substitution as a particularly attractive design direction for next-generation near-infrared-active DSSC materials.

## Figures and Tables

**Table 1:** Geometric parameters of the optimized dye-sensitized molecules.

Dyes	R1 (Å)	R2 (Å)	R3 (Å)	Φ1 ^a^ (°)	Φ2 ^a^ (°)	Φ3 ^a^ (°)
**XY1**	1.469	1.451	1.436	34.2	9.9	0.4
**HJ101**	1.468	1.449	2.769	42.3	0.3	0.7
**HJ102**	1.469	1.449	1.436	47.4	16.0	0.1
**HJ103**	1.482	1.449	1.437	79.8	0.7	0.1
**HJ104**	1.483	1.445	1.448	85.3	26.3	0.1
**HJ105**	1.433	1.445	1.437	35.8	2.4	0.4
**HJ106**	1.431	1.444	1.437	42.2	8.2	0.1

^a^ The range of dihedral angles measured is 0° to 90°.

**Table 2:** Excitation energies and major molecular orbital transitions obtained from TDDFT calculations.

Molecule	State ^(a)^	∆*E_exc_* ^(b)^ (eV)	*λ_osc_* ^(c)^ (nm)	*F* ^(d)^	Orbital Contribution (%) ^(e)^
**XY1**	S_1_	2.32	534	2.3534	H→L 53.6%, H−1→L 23.6%, H→L+1 12.1%
**HJ101**	S_1_	2.39	518	2.4610	H→L 50.4%, H−1→L 32.1%, H→ L+1 6.8%
**HJ102**	S_1_	1.81	684	1.2232	H→L 82.8%, H−1→L 9.0%
**HJ103**	S_1_	2.48	499	2.3531	H−1→L 81.1%, H−5→L+1 5.7%
**HJ104**	S_1_	2.00	621	1.2632	H−1→L 83.9%, H→L 6.1%
**HJ105**	S_1_	2.06	602	3.1029	H→L 69.6%, H−1→L 12.3%
**HJ106**	S_1_	1.56	795	1.8634	H→L 87.9%

^(a)^ This paper examines only excited states within the 400–800 nm wavelength range; when this range contains multiple excited states, the state with the highest oscillator strength is selected as the representative state. ^(b)^ The table lists the excitation energies of the corresponding excited states. ^(c)^ The table shows the absorption wavelengths corresponding to electronic transitions with nonzero oscillator strength. ^(d)^ Oscillator strength data are listed in the corresponding columns of the table. ^(e)^ Orbital pairs contributing less than 5% to the transition are not listed; H denotes the HOMO, and L denotes the LUMO. The arrows indicate transitions from occupied orbitals to vacant orbitals; e.g., H→L denotes HOMO→LUMO.

**Table 3:** Photovoltaic performance metrics of dye-sensitizer molecules.

Molecule	*λ_max_* (nm)	*ε* (10^4^·M^−1^·cm^−1^)	*LHE*	*τ* (ns)
**XY1**	534	9.6756	0.996	1.819
**HJ101**	518	10.0424	0.997	1.634
**HJ102**	680	9.0837	0.940	5.667
**HJ103**	498	9.6528	0.996	1.580
**HJ104**	616	5.2286	0.945	4.503
**HJ105**	601	12.6352	0.999	1.745
**HJ106**	795	7.5539	0.986	5.084

**Table 4:** Photovoltaic Parameters in DSSCs Calculated using TDDFT.

Molecule	∆*G_inj_* (eV)	∆*G_reg_* (eV)
**XY1**	−1.87	−1.50
**HJ101**	−1.81	−1.63
**HJ102**	−1.41	−1.47
**HJ103**	−1.80	−1.74
**HJ104**	−1.39	−1.70
**HJ105**	−1.50	−1.63
**HJ106**	−1.16	−1.43

## Data Availability

The data will be made available upon reasonable request from the corresponding author.

## Supplementary Materials

The following supporting information can be downloaded at: https://www.mdpi.com/article/10.3390/ijms27177646/s1.

## Abbreviations

The following abbreviations are used in this manuscript:

DSSCs	dye-sensitized solar cells
NIR	near-infrared
DFT	density functional theory
TDDFT	time-dependent density functional theory
HOMO	highest occupied molecular orbital
LUMO	lowest unoccupied molecular orbital
ICT	intramolecular charge transfer
CT	charge transfer
TDM	transition density matrix
LHE	light-harvesting efficiency
*V_oc_*	open-circuit voltage
*J_sc_*	short-circuit current density
*FF*	fill factor
*P_inc_*	incident light power intensity
*η*	power conversion efficiency
∆*G_inj_*	electron injection driving force
∆*G_reg_*	dye regeneration driving force
IEFPCM	integral equation formalism polarizable continuum model
BTD	benzothiadiazole
DFBTD	5,6-difluoro-2,1,3-benzothiadiazole
PBE0	Perdew–Burke–Ernzerhof hybrid exchange-correlation functional (0-parameter hybrid variant)
CAM-B3LYP	Coulomb-attenuating method Becke, 3-parameter, Lee-Yang-Parr functional
