# Supplementary material for: Theoretical Design of Near-Infrared-Absorbing D-A-π-A Dyes with Modified Hagfeldt Donors: A DFT/TDDFT Study
Source: Int J Mol Sci. 2026 Aug 26;27(17):7646. doi: 10.3390/ijms27177646 (PMC13566867; doi:10.3390/ijms27177646)
Supplement: Supplementary file 1 [file ijms-27-07646-s001.zip › ijms-4532731-supplementary.pdf]

Supporting Information

Theoretical Design of Near-Infrared-Absorbing D-A- π -A Dyes with Modified Hagfeldt Donors: A DFT/TDDFT Study

Jing Huang[1,2,a,*], Zhixiang Hu[3,b]

[1]Fujian Provincial Key Laboratory of Ecological Impacts and Treatment Technologies for Emerging Contaminants, College of Environmental and Biological Engineering, Putian University, Putian, Fujian 351100, China

[2]Key Laboratory of Ecological Environment and Information Atlas, Fujian Provincial University, Putian, Fujian 351100, China

[3]Strait Institute of Flexible Electronic, Fujian Normal University, Fuzhou, Fujian, 350117, China

[a] jinghuang@ptu.edu.cn

*corresponding author

SUMMARY

S1. Principle of transition density matrix······S2

S2. Fragmentation scheme of TDM analysis······S3

S3. Frontier molecular orbitals······S4

S4. UV-Vis spectroscopy······S4

S5. Heat maps of fragment transition density matrix······S7

S6. DFT optimized cartesian coordinates······S7

S1. Principle of transition density matrix

The transition density matrix (TDM) is a central quantity in quantum chemistry that characterizes the electronic transition between two many-electron quantum states—typically the ground state and an excited state. Unlike the one-particle reduced density matrix (1-RDM), which describes the electron distribution within a single quantum state, the TDM encodes the single-electron properties associated with the transition process itself, such as atomic transition charges, transition electric and magnetic dipole moments, and related observables.

The TDM can be expressed in two common representations: the real-space form and the matrix form in a basis-function representation. These two forms offer complementary advantages for theoretical analysis and visualization.

S1.1 Real-Space Representation of the TDM

For an N-electron system, the real-space TDM between the ground state Φ_0 and an excited state Ψ_{exc} is defined as:

$$T(r;r')=N\int\Phi_0^*(x_1,...,x_N)\,\Psi_{exc}(x_1,...,x_N)d\sigma_1 dx_2...dx_N$$

where x=(r,σ) denotes the combined spatial and spin coordinates of an electron, and N is the total number of electrons. This expression is termed the “real-space form” because its two arguments, r and r′ , are three-dimensional spatial coordinates, rendering T(r;r′) a six-dimensional function.

In single-reference excited-state methods—such as time-dependent density functional theory (TDDFT), configuration interaction singles (CIS), or the ZINDO method, the excited-state wavefunction is constructed as a linear combination of singly excited configuration state functions.

S1.2 Matrix Representation of the TDM in a Basis-Function Framework

When molecular orbitals are expanded in a finite set of atomic orbital (AO) basis functions $\{\chi_\mu(r)\}$, the TDM assumes a discrete matrix form indexed by basis-function labels μ and ν:

$$T_{\mu\nu}=\sum_{i,a}\omega_{ia}C_{\mu i}^*C_{\nu a}$$

$$T(r;r')=\sum_{\mu,\nu}\chi_\mu^*(r')T_{\mu\nu}\chi_\nu\,(r)$$

where $C_{\mu i}$ and $C_{\nu a}$ are the expansion coefficients of the occupied orbital i and virtual orbital a, respectively, in the AO basis. The matrix form is particularly useful in sum-over-states (SOS) approaches and for computing properties that depend on transition amplitudes between multiple excited states.

A common visualization technique is the heat map, in which each matrix element $T_{\mu\nu}$ is represented by a colored tile whose hue and intensity reflect the magnitude and sign of the element. While informative, this representation is often of limited chemical interpretability because individual basis functions lack direct physical meaning.

S2. Fragmentation Scheme of TDM analysis

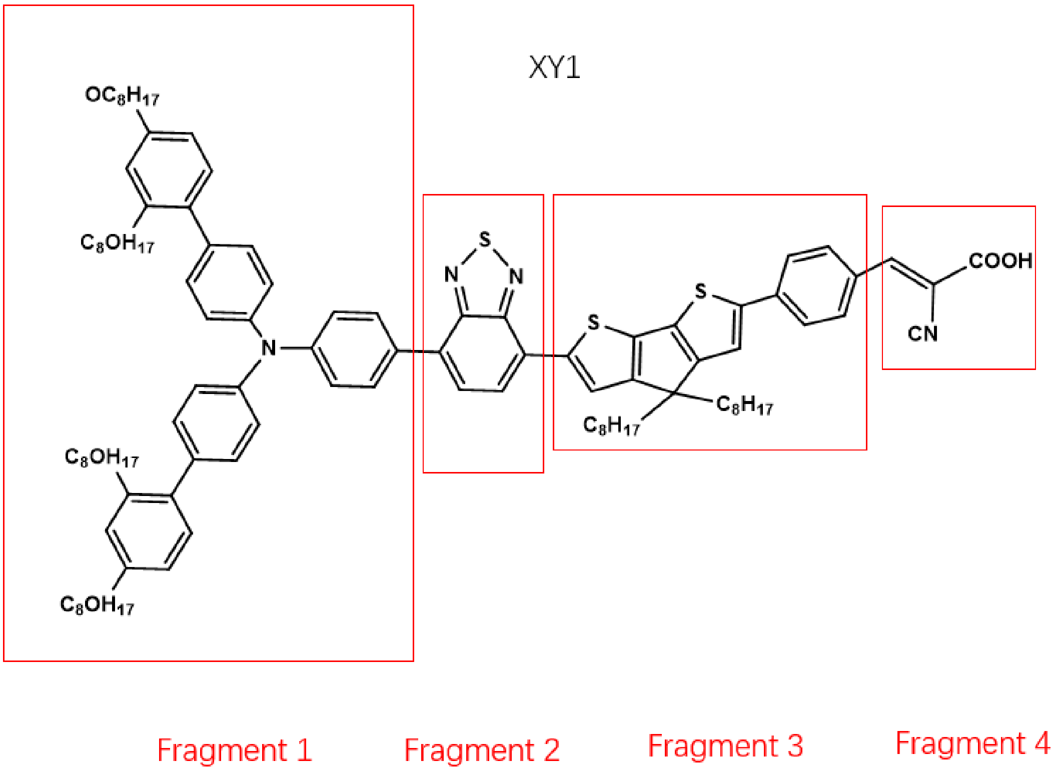

Figure S1. The fragmentation scheme of XY1, HJ101~HJ106.

S2. Frontier molecular orbitals

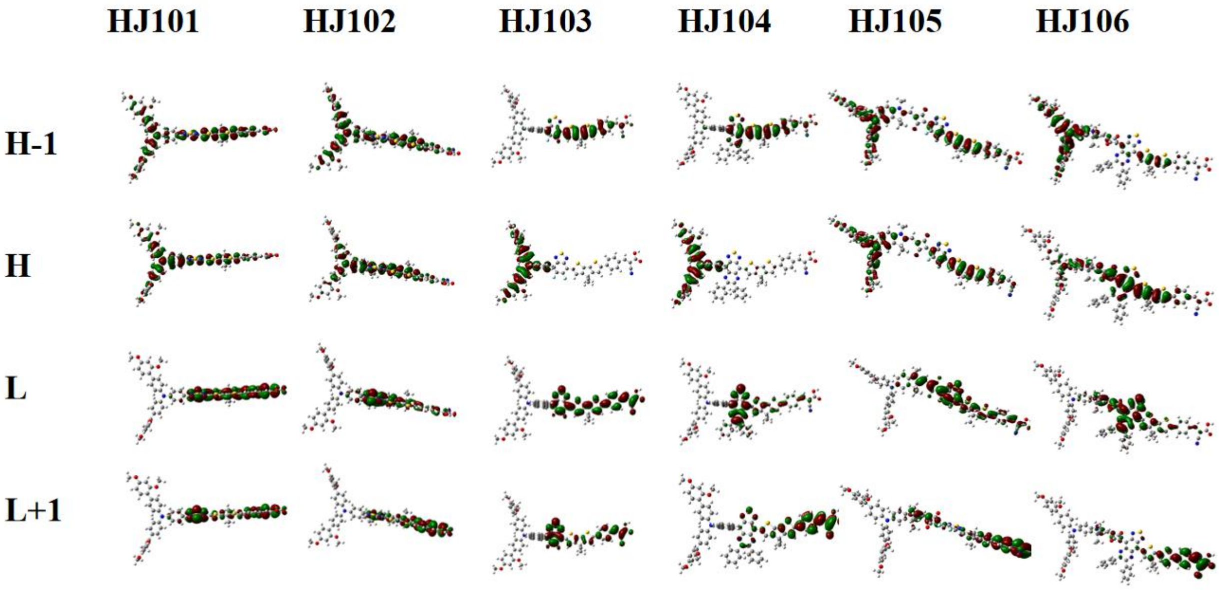

S3. UV-Vis spectroscopy

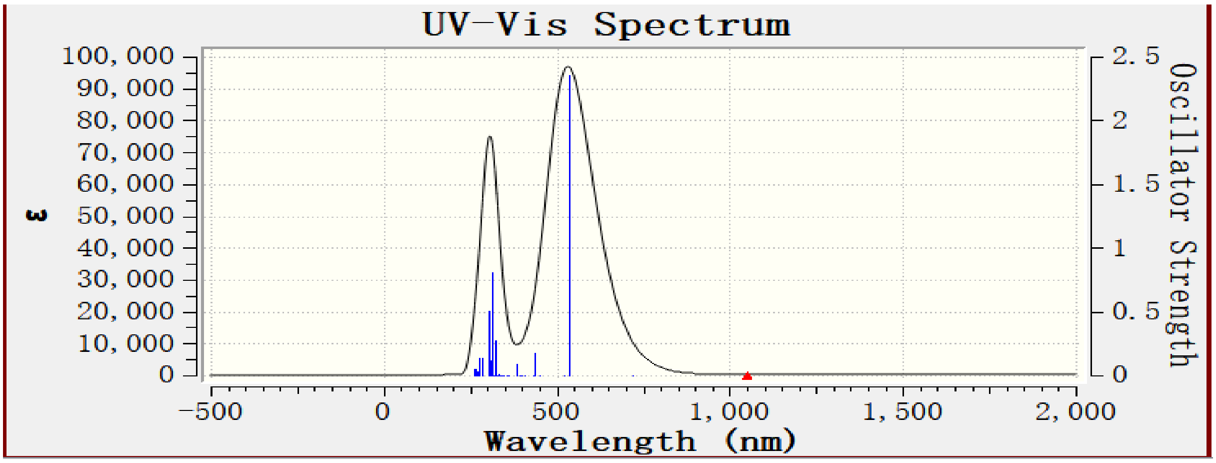

XY1

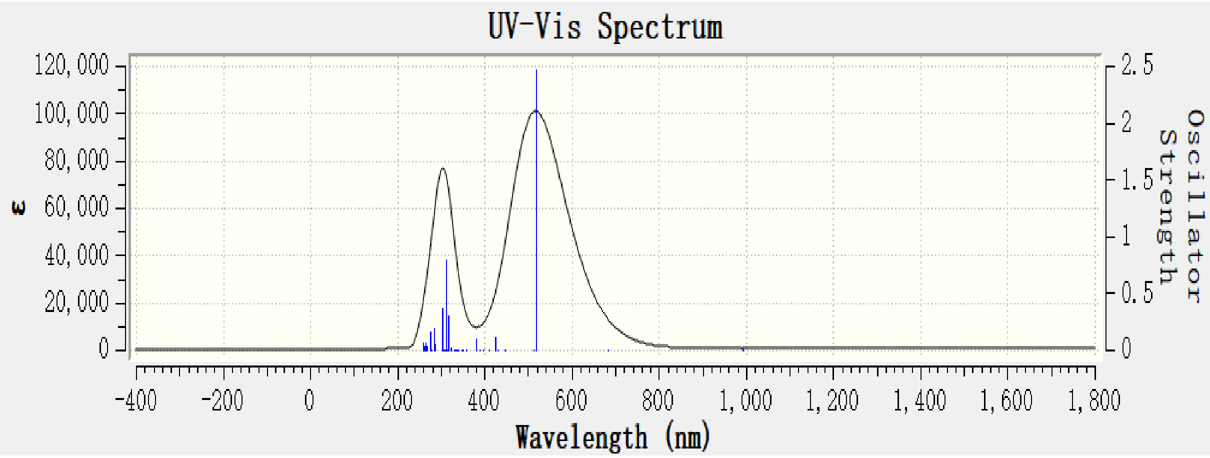

HJ101

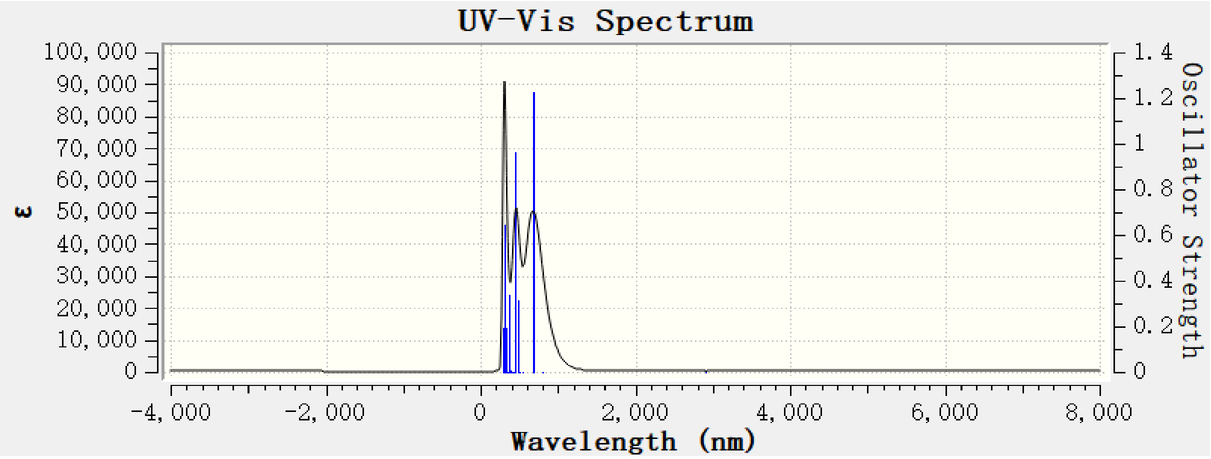

HJ102

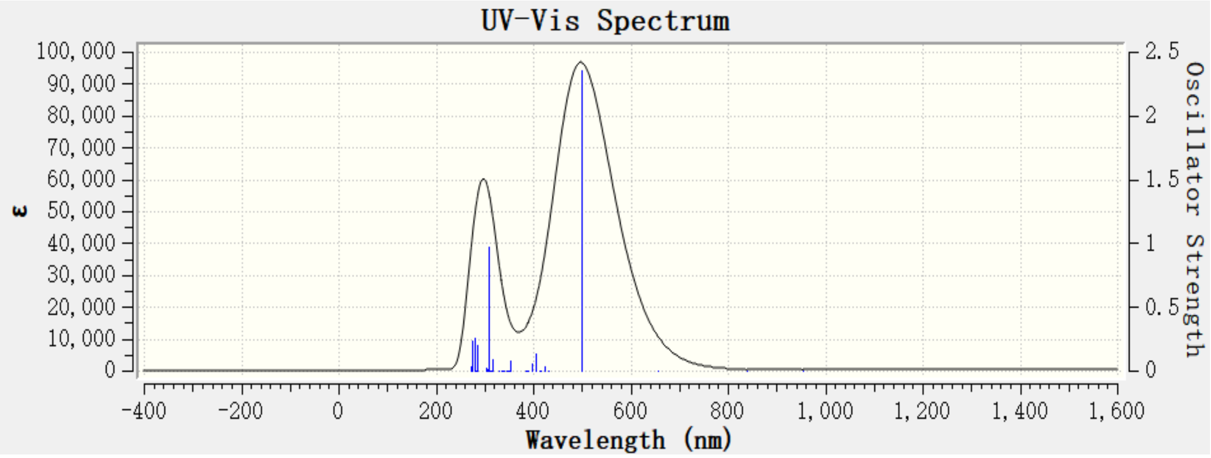

HJ103

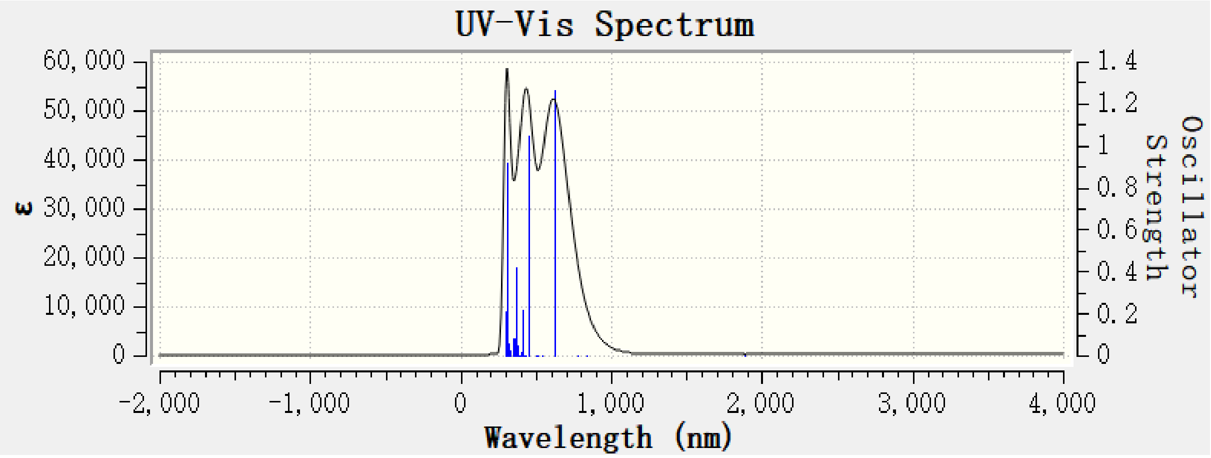

HJ104

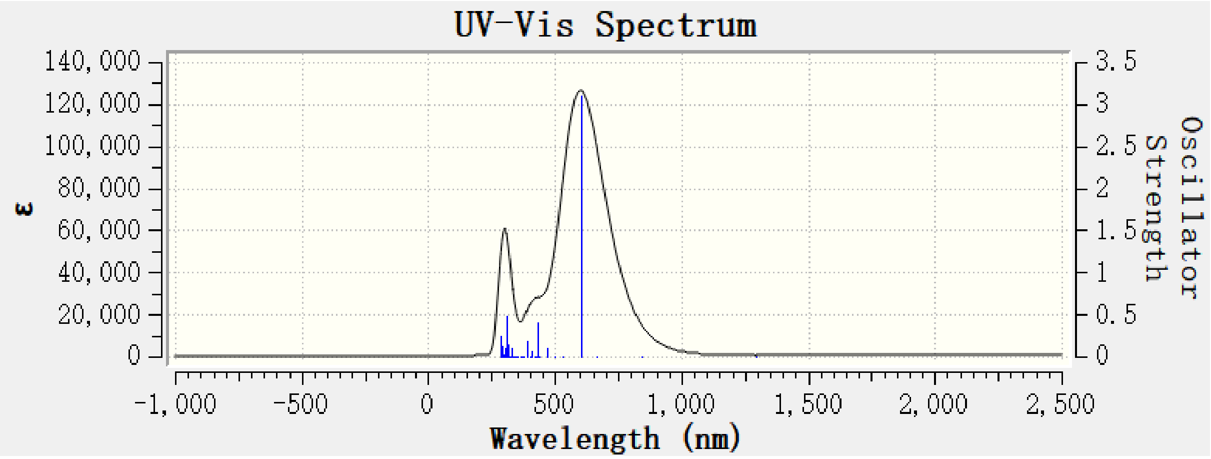

HJ105

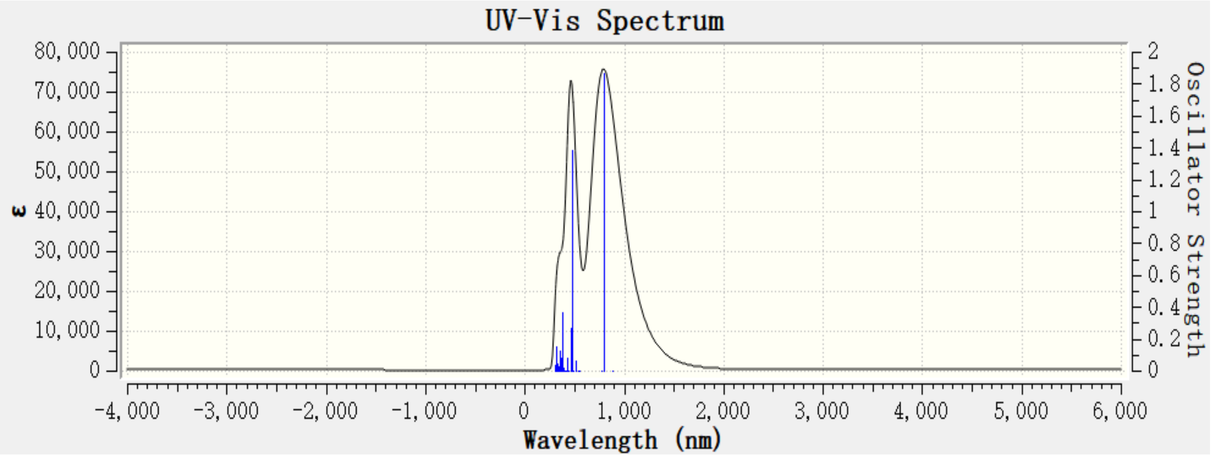

HJ106

Figure S2. The TDDFT calculated UV-Vis spectroscopy ofXY1, HJ102~HJ106.

S3. Heat maps of fragment transition density matrix

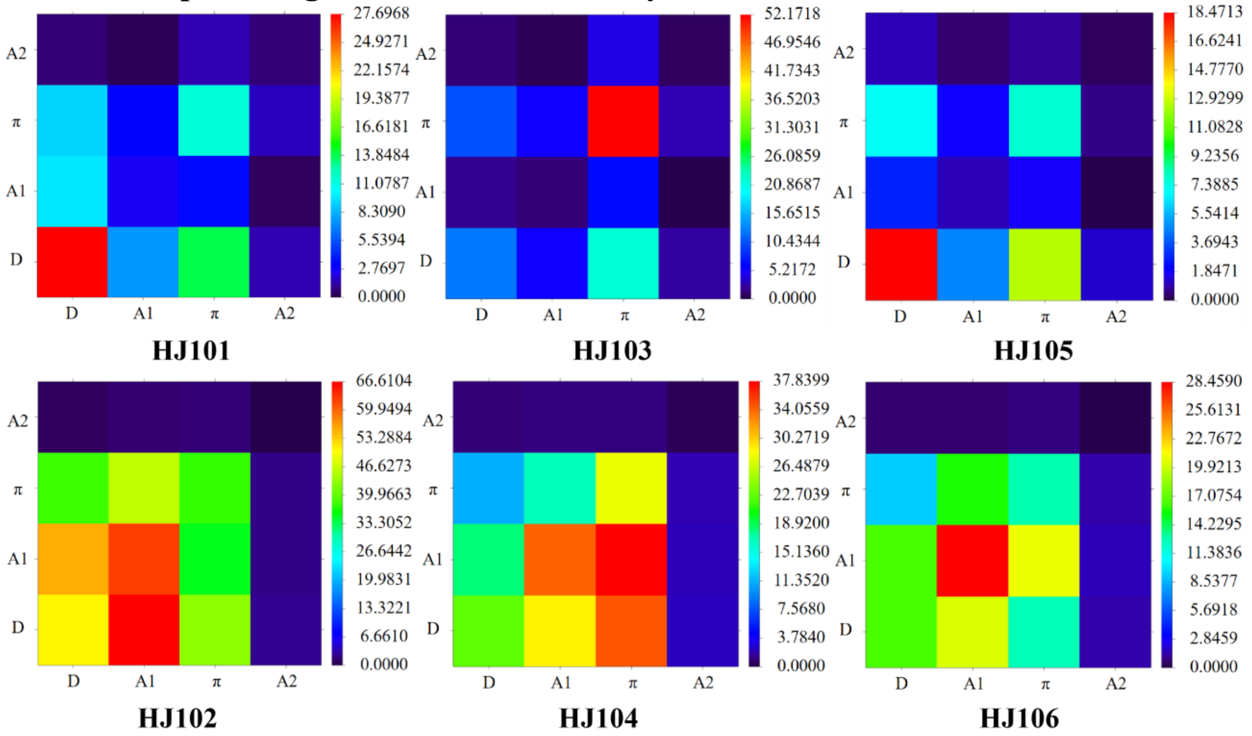

Figure S3. fragment transition density matrix heatmaps of HJ101~HJ106

S4. DFT optimized cartesian coordinates

XY1

N	6.12281446	0.01375400	0.10777001
C	0.42172503	0.05982200	0.26600302
C	-0.24955202	0.51214804	1.38369010
C	-0.41831803	-0.38279303	-0.80394606
C	-1.65464513	0.54339504	1.49713611
H	0.32686902	0.84978806	2.23864317
C	-1.86056014	-0.36097503	-0.68528605
C	-2.50969919	0.10529101	0.50224204
H	-2.06835016	0.93019907	2.42135618
C	6.82891153	-1.21117509	0.14272601
C	7.93025359	-1.41885011	-0.69209105
C	6.44810151	-2.23358917	1.01363908
C	8.63114467	-2.61383220	-0.65272205
H	8.24049465	-0.63221205	-1.37187311
C	7.14687652	-3.43181126	1.03656408
H	5.59497343	-2.09188116	1.66864413
C	8.25688163	-3.64950128	0.21221902
H	9.48318571	-2.74720221	-1.30878810
H	6.82128254	-4.21727732	1.71157313
C	6.84894253	1.22583809	0.05192800
C	7.98136258	1.41219411	0.84663307
C	6.45475448	2.25750217	-0.80469006
C	8.69791668	2.59814720	0.77941606
H	8.29652764	0.62533005	1.52377312
C	7.16703055	3.44532426	-0.85348107
H	5.58567341	2.12234116	-1.43998511
C	8.30944264	3.64212428	-0.06788401
H	9.56461773	2.72640621	1.42061911
H	6.84413655	4.22626532	-1.53186312
C	9.01663268	-4.91493037	0.30637402
C	9.32458373	-5.45445840	1.55124512
C	9.44744270	-5.63578242	-0.83163906
C	10.02774874	-6.64591649	1.71319013
H	9.01701670	-4.91034038	2.43893219
C	10.14988078	-6.82523550	-0.69257305
C	10.44344679	-7.33098554	0.57690804
H	10.24622078	-7.00893054	2.70896320
H	10.47995282	-7.39272756	-1.55313812
C	9.12064167	4.87550737	-0.14779001
C	8.54871664	6.16899745	-0.14079301
C	10.50785983	4.79656137	-0.21761202
C	9.35261070	7.29888057	-0.20765502
C	11.33260485	5.91709146	-0.28535502
H	10.96725082	3.81315129	-0.23949102
C	10.74302180	7.17622453	-0.28307102
H	8.93156468	8.29590163	-0.19575801
H	12.40539393	5.79075044	-0.34955003

O 9.11654669 -5.11884239 -2.03743915
O 7.20095156 6.23488345 -0.03909900
O 11.13755387 -8.49425266 0.59091505
O 11.42497888 8.34479162 -0.35077303
C 11.45831487 -9.05910968 1.84883414
H 10.55460581 -9.30030169 2.41835118
H 12.00563994 -9.97615777 1.63572513
H 12.09234092 -8.38693562 2.43652819
C 9.49743371 -5.82481443 -3.20315724
H 10.58636983 -5.91417146 -3.27742425
H 9.04570970 -6.82208753 -3.23010725
H 9.12595869 -5.23796240 -4.04205131
C 6.58822352 7.50969055 0.00366300
H 6.92723754 8.08557859 0.87131806
H 6.78454150 8.07771163 -0.91182707
H 5.51855140 7.32384456 0.08986701
C 12.83752800 8.28405165 -0.42474703
H 13.16612903 7.75516857 -1.32561910
H 13.18086403 9.31663570 -0.46765704
H 13.26150799 7.79753562 0.46001403
N -0.00760700 -0.82233106 -1.99519715
S -1.33034310 -1.17979309 -2.86100122
N -2.47975419 -0.79379106 -1.78152714
C -7.83208761 0.07319401 0.28989802
C -8.35722062 0.32768702 1.54702412
C -6.40398347 0.08895901 0.36722303
C -7.25007254 0.52975404 2.56570920
C -6.03388648 0.34982103 1.67521013
C -3.95135630 0.11845801 0.66963605
C-10.30681978 0.08592301 0.30808302
C -4.64424535 0.36522703 1.84630314
C -9.75710675 0.33472403 1.55688412
S -5.05889039 -0.15203401 -0.66558405
S -9.04784768 -0.15945301 -0.89056707
C -11.70333288 0.01272000 -0.06239400
C -12.10651795 -0.17598501 -1.39679610
C -12.71567198 0.12925501 0.91294007
C -13.44127305 -0.24435402 -1.73163613
H -11.36405486 -0.26713502 -2.18292317
C -14.04936809 0.06394300 0.57827004
H -12.44938697 0.26875502 1.95392715
C -14.45321309 -0.12589501 -0.75899206
H -13.72138403 -0.39085403 -2.77025121
H -14.78465411 0.15810901 1.36674810
C -15.81135920 -0.21162502 -1.21818109
H -15.91032620 -0.36627303 -2.28899618
C -17.00336530 -0.13508301 -0.55914404
C -17.14130932 0.06536401 0.84033306
N -17.26787629 0.22852702 1.97790215
C -18.29065241 -0.25829502 -1.27405410
O -19.36968546 -0.19695502 -0.73103705
O -18.14861340 -0.44631903 -2.59193020
H -19.03424646 -0.51488804 -2.97303823
H -4.14734032 0.53681304 2.79304922
H -10.36009078 0.51828404 2.43760919
C -7.29966253 -0.53751904 3.66695428
H -6.45097251 -0.42069503 4.34702133
H -8.21936862 -0.43962003 4.25082833
H -7.26553558 -1.54313012 3.24038725
C -7.29762457 1.93717615 3.17474724
H -8.21618562 2.07097616 3.75339529
H -6.44764647 2.08886916 3.84623429
H -7.26434757 2.70306221 2.39583918
C 4.02312031 1.04842508 0.79305706
C 2.63928820 1.05733908 0.81299306
C 1.88855915 0.05143800 0.18931001
C 2.59452420 -0.96430607 -0.46991004
C 3.97846030 -0.97962507 -0.49670804
C 4.71976136 0.02646300 0.13573501
H 4.57290635 1.84358714 1.28402110
H 2.13108516 1.87336815 1.31671810
H 2.05123116 -1.75569913 -0.97150908
H 4.49535734 -1.78029414 -1.01398808

HJ101

N 6.15556700 0.00685900 0.10991800
C 0.47117900 -0.17963100 0.43461700
C -0.21015700 -0.50616300 1.57786400
C -0.38678300 0.12043400 -0.66966400
C -1.62106100 -0.54836200 1.69153300
C -1.82780300 0.07838000 -0.55673500
C -2.49855600 -0.26445200 0.66677300
C 6.90946300 -1.18915100 0.03824800
C 6.52272900 -2.22927100 -0.81100900
C 8.06542100 -1.34401800 0.80437900
C 7.26848900 -3.39535400 -0.88266300
H 5.62821400 -2.12233400 -1.41567600
C 8.81390100 -2.50908400 0.71641800
H 8.38374400 -0.54294400 1.46319100
C 8.43165800 -3.56339900 -0.12091000
H 6.94522600 -4.18823300 -1.54689400
H 9.71730100 -2.60035100 1.31146300
C 6.83477100 1.24761300 0.08279600
C 7.88578500 1.46057800 -0.81062600
C 6.47653400 2.27932500 0.95469900
C 8.55816000 2.67397700 -0.82583200
H 8.17185300 0.67331900 -1.50011900
C 7.14326300 3.49406500 0.92203800
H 5.67262600 2.12386900 1.66656700
C 8.20399800 3.71890700 0.03551200

H 9.36035900 2.82164000 -1.54230600
H 6.85020700 4.27476500 1.61412100
C 9.21746000 -4.81617000 -0.14631700
C 9.65484800 -5.39099900 1.04282400
C 9.56976000 -5.47371400 -1.34785400
C 10.40158200 -6.56579600 1.09175500
H 9.38479000 -4.91010900 1.97796400
C 10.31327700 -6.64602300 -1.32143000
C 10.72737500 -7.19548400 -0.10454200
H 10.70322500 -6.97041200 2.04895900
H 10.59708100 -7.15748100 -2.23218200
C 8.96973400 4.98368900 0.02295300
C 8.34921200 6.25449100 0.05104100
C 10.35974500 4.95844800 -0.03615000
C 9.11106800 7.41495900 0.02659100
C 11.14263400 6.11026600 -0.06183900
H 10.85792400 3.99388600 -0.04422800
C 10.50632500 7.34619700 -0.02678600
H 8.65106200 8.39464000 0.03962900
H 12.22084200 6.02552500 -0.09713000
O 9.16942200 -4.88713400 -2.49953300
O 6.99645000 6.26839000 0.07337700
O 11.44104300 -8.34278800 -0.19962300
O 11.14545300 8.54030700 -0.04042500
C 11.89282600 -8.94422100 1.00089300
H 11.05288700 -9.22932200 1.64280600
H 12.43806000 -9.83823600 0.70213700
H 12.56410600 -8.27694100 1.55132600
C 9.52162500 -5.49802800 -3.72740600
H 10.60868300 -5.55172900 -3.84766200
H 9.09391800 -6.50261800 -3.80959700
H 9.10336900 -4.86319100 -4.50717200
C 6.33314600 7.51931100 0.07481500
H 6.59247900 8.10663800 0.96178300
H 6.56724400 8.09488300 -0.82684000
H 5.26817600 7.29222400 0.09146000
C 12.56076000 8.53405400 -0.10241400
H 12.91599000 8.04797600 -1.01697900
H 12.86576400 9.57939300 -0.10748300
H 12.99439400 8.03405400 0.76984200
N 0.02633800 0.43697700 -1.89462100
S -1.29498000 0.67102200 -2.80565700
N -2.44313100 0.37384900 -1.69564700
C -7.80586000 -0.17883300 0.28746800
C -8.37904600 -0.51402000 1.50373100
C -6.38157800 -0.21120000 0.41848700
C -7.31364700 -0.79948200 2.54638500
C -6.06392000 -0.56805700 1.71662300
C -3.94046900 -0.30874600 0.80442500
C -10.27801400 -0.16282400 0.21462100
C -4.68352300 -0.62397800 1.93778300
C -9.77885700 -0.50456800 1.46231400
S -5.00061700 0.06117400 -0.55362000
S -8.97442800 0.15018300 -0.91777800
C -11.66036100 -0.04782300 -0.20037600
C -12.01178800 0.25519100 -1.52794700
C -12.70808700 -0.23688300 0.72451800
C -13.33299300 0.36410000 -1.90459700
H -11.24039300 0.40599600 -2.27611300
C -14.02811700 -0.13095100 0.34794600
H -12.48190500 -0.46667300 1.75886300
C -14.38037800 0.17445200 -0.98221900
H -13.57337700 0.59958700 -2.93675700
H -14.79227800 -0.28406200 1.09880700
C -15.72040200 0.31303100 -1.48103600
H -15.77895800 0.56322500 -2.53654700
C -16.93590300 0.18903500 -0.87465700
C -17.12750800 -0.13598300 0.49461500
N -17.30231000 -0.40036500 1.60656300
C -18.19627500 0.38866200 -1.62033100
O -19.29402500 0.28586600 -1.12116900
O -18.00663900 0.69600000 -2.90757500
H -18.87635000 0.80777100 -3.31474100
H -4.23080500 -0.88254000 2.88297100
H -10.41554000 -0.74089600 2.30576200
C -7.39442500 -2.24687900 3.04931900
H -6.57302100 -2.45384100 3.74116200
H -8.33649100 -2.41239900 3.57973500
H -7.33497600 -2.95499000 2.21892200
C -7.39800900 0.18532200 3.72007400
H -8.33856500 0.05127800 4.26185300
H -6.57494100 0.01235900 4.41919100
H -7.34374400 1.21920000 3.36985200
C 4.11137800 -1.08220800 0.86676200
C 2.73036500 -1.12660200 0.94317300
C 4.75752300 -0.03924100 0.18984700
C 1.93682700 -0.13652700 0.34988700
C 2.58807000 0.90160600 -0.32841800
C 3.96856400 0.95332600 -0.40695200
F -2.06785000 -0.87914400 2.90634500
F 0.45081300 -0.78365400 2.70663700
H 2.26327200 -1.95005800 1.47144500
H 4.69882900 -1.86341200 1.33566800
H 2.00495700 1.68022200 -0.80568500
H 4.44402400 1.76929200 -0.93930300

HJ102

N 6.20658300 0.14609700 -0.93326500
C 0.54094900 0.20186200 -0.29198500
C -0.11858400 -0.05177500 0.92112200
C -0.30795500 0.48009200 -1.38672200

C -1.55814600 -0.03576200 1.05341000
C -1.75144600 0.50433900 -1.26121100
C -2.42248200 0.25479400 -0.03167200
C 6.90192900 -1.07442100 -1.09596100
C 6.41714900 -2.06514200 -1.95445100
C 8.09231100 -1.31111000 -0.40611400
C 7.09808700 -3.26281000 -2.10627900
H 5.50136600 -1.89056200 -2.50945300
C 8.77591300 -2.50623700 -0.57612400
H 8.47900700 -0.55718900 0.27142300
C 8.29677900 -3.51034600 -1.42552600
H 6.70358100 -4.01093600 -2.78380900
H 9.68971100 -2.67380200 -0.01430400
C 6.93778800 1.35635800 -0.90490700
C 7.96866900 1.58311800 -1.81842700
C 6.65604500 2.34249200 0.04484500
C 8.69807400 2.76255000 -1.77513700
H 8.20577300 0.82616900 -2.55855600
C 7.38041400 3.52381500 0.07206900
H 5.85967200 2.18118400 0.76377400
C 8.41926200 3.76251000 -0.83640000
H 9.50456600 2.90845000 -2.48709800
H 7.13956300 4.27267300 0.81747100
C 9.07163700 -4.75526100 -1.61488400
C 10.45085900 -4.70054600 -1.79075700
C 8.47346200 -6.03703100 -1.60825000
C 11.24300500 -5.83351600 -1.96175700
H 10.92929100 -3.72626300 -1.81354500
C 9.24482000 -7.17910900 -1.77691000
C 10.62772700 -7.08048400 -1.95650900
H 12.31014900 -5.72566000 -2.10496500
H 8.80367200 -8.16749500 -1.76567900
C 9.17691200 5.03265900 -0.85475800
C 9.68444200 5.63796800 0.31847200
C 9.43157600 5.67718000 -2.06130900
C 10.39674700 6.82787700 0.24999600
C 10.14393100 6.87071500 -2.15243100
H 9.04030000 5.23712500 -2.97340800
C 10.62509500 7.44748000 -0.98215700
H 10.79793700 7.29997600 1.13760900
H 10.30029200 7.32978100 -3.11980900
O 7.13685600 -6.08087400 -1.40279300
O 9.46116600 4.98520900 1.48250700
O 11.27691000 -8.25851200 -2.11662600
O 11.32552100 8.60560800 -0.92755800

C 12.68202300 -8.22249200 -2.29352900
H 13.18009500 -7.77565200 -1.42683200
H 12.99839900 -9.25935800 -2.39661500
H 12.95458100 -7.66863400 -3.19786000
C 6.50025700 -7.34480800 -1.36026700
H 6.61375700 -7.88026500 -2.30861200
H 6.89134900 -7.96001600 -0.54320500
H 5.44477900 -7.14141700 -1.18527600
C 9.97261200 5.54267900 2.67928300
H 11.06461700 5.61752000 2.64858600
H 9.54309000 6.53092300 2.87379700
H 9.68020000 4.85951700 3.47539700
C 11.59234600 9.27709300 -2.14610000
H 10.66521300 9.57381600 -2.64737600
H 12.15963900 10.16777000 -1.88024200
H 12.18954200 8.65432000 -2.82016300
N 0.10625500 0.71519600 -2.64202800
S -1.21068700 0.95661200 -3.54048600
N -2.36098500 0.77590600 -2.42127900
C -7.72014600 0.26725800 -0.58423000
C -8.33288900 0.36194500 0.65649400
C -6.30232000 0.26929600 -0.40749500
C -7.30057900 0.44544700 1.76574000
C -6.02534800 0.37365300 0.94571700
C -3.86713300 0.30331100 0.07145100
C -10.18980400 0.25639000 -0.73373200
C -4.65543400 0.38922400 1.21740700
C -9.72974000 0.35519100 0.57137500
S -4.88974800 0.22482900 -1.36999800
S -8.85053100 0.16801800 -1.86480500
C -11.55780100 0.22380700 -1.20379900
C -11.87042500 0.22361200 -2.57536000
C -12.63222800 0.19229800 -0.29025600
C -13.17974300 0.19544100 -3.00398200
H -11.07758900 0.25089100 -3.31579200
C -13.94027700 0.16694600 -0.71829700
H -12.43563300 0.18196900 0.77516800
C -14.25373500 0.16833900 -2.09281200
H-13.39016000 0.19739000 -4.06900600
H -14.72596000 0.14267700 0.02556000
C -15.57796800 0.14423100 -2.64793800
H -15.60566000 0.14945200 -3.73394900
C -16.81089800 0.11677400 -2.06429900
C -17.04231500 0.10567000 -0.66316500
N -17.24904900 0.09636200 0.47445000
C -18.04847100 0.09665700 -2.87140000
O -19.16046500 0.07322300 -2.39386400
O -17.82166800 0.10699100 -4.18913600
H -18.67938200 0.09271000 -4.63504100
H -4.21355400 0.44728400 2.19987600
H -10.39302900 0.42817500 1.42428000
C -7.41571700 -0.74147900 2.73125700
H -6.61423400 -0.70333800 3.47476300
H -8.37285400 -0.71058100 3.25976600

H -7.34606400 -1.69159900 2.19547300
C -7.40303500 1.77274600 2.52888200
H -8.35973500 1.83591700 3.05535400
H -6.60104600 1.84617500 3.26889400
H -7.32500100 2.62408300 1.84790800
C 4.15736400 -0.88583800 -0.10423500
C 2.78308000 -0.86860800 0.05775200
C 4.81187700 0.15462000 -0.77661400
C 2.00175300 0.17903700 -0.44764100
C 2.66305500 1.20866500 -1.12996600
C 4.03846600 1.20392100 -1.28856100
C 1.34720400 -0.53144900 3.30939900
C 0.09498700 -0.58758500 3.16545100
N -2.09924700 -0.28556600 2.27310500
N 0.64649400 -0.32822300 2.01474400
C -2.05398300 -0.70093600 4.59977800
C -3.22666800 -1.46116000 4.64210500
C -1.61904600 -0.05485200 5.76083600
C -3.93883100 -1.59084500 5.82739000
H -3.56833100 -1.95546600 3.73900400
C -2.34407500 -0.17017800 6.94014300
H -0.72204300 0.55459200 5.73963000
C -3.49991600 -0.94452000 6.97939300
H -4.83904800 -2.19630300 5.85168400
H -2.00527200 0.34777300 7.83130200
H -4.05839100 -1.04150300 7.90466700
C 1.01393700 -0.96638100 4.26440600
C 0.76098300 -2.07930300 5.07128500
C 2.19425700 -0.24225200 4.45207700
C 1.67278000 -2.45746400 6.04886700
H -0.14227900 -2.66196900 4.92485300
C 3.09457300 -0.61016000 5.44364200
H 2.39498500 0.61452200 3.81781100
C 2.83661300 -1.71958600 6.24374600
H 1.47347100 -3.33171500 6.65978300
H 4.00094200 -0.03185200 5.59029300
H 3.54236100 -2.01073200 7.01487300
H 4.73374700 -1.71197900 0.29692900
H 2.30546500 -1.68317400 0.58818100
H 2.09051600 2.03241000 -1.54071000
H 4.52096200 2.01963500 -1.81537100

HJ103

N -5.99028700 0.10590300 0.06029000
C -0.28248700 -0.38146300 0.21901800
C 0.37090700 -1.43313300 0.79080600
C 0.57680300 0.60945900 -0.33350700
C 1.78310300 -1.56249700 0.84454200
C 2.01427900 0.48501400 -0.28318400
C 2.66884600 -0.64045800 0.32876000
C -6.55147600 1.39807300 0.07480700
C -5.85811000 2.47109700 -0.49586600
C -7.78256500 1.65647100 0.68687700
C -6.38062500 3.75487900 -0.45812700
H -4.89679500 2.29807300 -0.96790400
C -8.30100900 2.94249400 0.70728300
H -8.33853100 0.84832100 1.14872000
C -7.61571500 4.02395000 0.14149100
H -5.81544700 4.56422900 -0.90558100
H -9.26525300 3.10901900 1.17788600
C -6.77314800 -1.06369500 0.00517000
C -7.98956400 -1.10013900 -0.68507800
C -6.31987100 -2.24122500 0.60955600
C -8.72720300 -2.27233500 -0.74720400
H -8.35311000 -0.21170300 -1.18921000
C -7.05867300 -3.41188000 0.52735900
H -5.38007100 -2.23809700 1.15151500
C -8.28548000 -3.45612600 -0.14387500
H -9.65889300 -2.27377600 -1.30492000
H -6.68121300 -4.30457400 1.01216600
C -8.17013100 5.39189900 0.23601000
C -8.70960700 5.84730300 1.43494800
C -8.19487600 6.28467300 -0.86066900
C -9.25362500 7.12021800 1.59186900
H -8.68894700 5.18418700 2.29436200
C -8.73109200 7.55840800 -0.72566600
C -9.25779700 7.97958100 0.49882200
H -9.64901600 7.42010700 2.55354800
H -8.76290500 8.25082400 -1.55708000
C -9.12265600 -4.67373900 -0.19312300
C -8.59643300 -5.95515700 -0.48009700
C -10.49366900 -4.58855600 0.02938800
C -9.42629300 -7.06733700 -0.52821900
C -11.34409600 -5.69115700 -0.01488800
H -10.91762200 -3.61754100 0.26617500
C -10.79848400 -6.93917800 -0.29383800
H -9.03998100 -8.05286600 -0.75429000
H -12.40112800 -5.56167900 0.17736600
O -7.69800000 5.82073100 -2.03090700
O -7.26770500 -6.02357400 -0.72722400
O -9.74753100 9.24283800 0.51590700
O -11.50843600 -8.09123400 -0.36142500
C -10.29211600 9.72549800 1.73119500
H -9.53902100 9.73913600 2.52593800
H -10.62218200 10.74342600 1.52937000
H -11.14896200 9.12284600 2.04986600
C -7.72311100 6.67299400 -3.16106800
H -8.74815500 6.95191700 -3.42690000
H -7.13008300 7.57765600 -2.99108400
H -7.28220800 6.10090900 -3.97616700

C -6.70306400 -7.28207000 -1.04610800
H -6.82923100 -7.99512000 -0.22474100
H -7.14203800 -7.69539400 -1.96023400
H -5.64118400 -7.10069100 -1.20605700
C -12.90577600 -8.02219000 -0.13969800
H -13.39434800 -7.38083000 -0.88057900
H -13.27714600 -9.04064200 -0.24314800
H -13.13059700 -7.65484700 0.86700800
N 0.16667600 1.72480300 -0.93341200
S 1.49703400 2.53327500 -1.39223300
N 2.63779700 1.51098200 -0.85074300
C 7.97918100 -0.34466200 0.17480100
C 8.53904500 -1.46439000 0.76796900
C 6.55332200 -0.45320900 0.22761300
C 7.46312700 -2.40886100 1.27186500
C 6.22204000 -1.63969400 0.85729500
C 4.10949600 -0.78386300 0.39795000
C 10.45036800 -0.29913100 0.15464000
C 4.83960100 -1.82905900 0.95504500
C 9.93989600 -1.43941500 0.75474300
S 5.18417000 0.44892800 -0.25714400
S 9.16005800 0.75031800 -0.40216400
C 11.83772400 0.07137600 -0.03702100
C 12.20546200 1.35179700 -0.48594500
C 12.87067100 -0.85122100 0.22573800
C 13.53090200 1.68696200 -0.66179900
H 11.44433700 2.09735600 -0.69164600
C 14.19517800 -0.51513600 0.05460700
H 12.62805600 -1.85307000 0.55989100
C 14.56436000 0.76795800 -0.39639800
H 13.78536600 2.68407700 -1.00744100
H 14.94873800 -1.26185400 0.26835800
C 15.91068600 1.22308700 -0.61146000
H 15.98323400 2.24939500 -0.96016900
C 17.11652700 0.60521900 -0.45911700
C 17.28998800 -0.73358900 -0.01744200
N 17.44990600 -1.82127700 0.34042900
C 18.38605100 1.30427300 -0.75146100
O 19.47595300 0.79278500 -0.62950100
O 18.21331900 2.56337200 -1.16579700
H 19.08758700 2.94143600 -1.33162700
H 4.37625000 -2.69023900 1.41205200
H 10.56955300 -2.21285500 1.17679900
C 7.53457900 -2.57710900 2.79555700
H 6.70447300 -3.19612900 3.14741100
H 8.46972200 -3.06759000 3.08013600
H 7.48310300 -1.60902500 3.30019200
C 7.54069600 -3.77287500 0.57362400
H 8.47870900 -4.27535900 0.82631900
H 6.71430800 -4.41203500 0.89731000
H 7.48834400 -3.66182400 -0.51238400
C -3.88526100 0.99135700 3.61125900
C -4.57002700 0.68306700 2.47165200
C -3.88472000 0.28410100 1.28735900
C -2.45227600 0.17794900 1.32168500
C -1.77724200 0.52644700 2.52933700
C -2.46858900 0.92358400 3.63664300
C -4.57377000 -0.02218300 0.10205600
C -1.75987600 -0.26877000 0.18401600
C -2.45332400 -0.59754300 -0.99229700
C -3.88155200 -0.45246900 -1.04227100
C -4.55575300 -0.73654200 -2.26546400
H -5.62997600 -0.60386000 -2.31190700
C -3.86945000 -1.16795100 -3.36344600
C -2.46324000 -1.34578700 -3.30608300
C -1.77926200 -1.06591900 -2.15908300
H -4.42412800 1.29006300 4.50428000
H -5.65196500 0.73693400 2.45437400
H -0.69450100 0.47160600 2.55914600
H -1.93482100 1.18386500 4.54463500
H -4.39926500 -1.37711300 -4.28683000
H -1.93092300 -1.70119200 -4.18212100
H -0.70364700 -1.20019300 -2.12413700
F 2.21523200 -2.67253700 1.44839100
F -0.31818600 -2.42998200 1.34811000

HJ104

N -6.06392900 0.84888700 -0.27761600
C -0.34605700 0.39833900 -0.24026400
C 0.28461700 -0.79550700 0.10298300
C 0.49908700 1.46523900 -0.59041600
C 1.72233900 -0.93816200 0.14087200
C 1.93795800 1.31355300 -0.63866400
C 2.58568500 0.09567200 -0.29043100
C -6.66291400 2.11527200 -0.14073900
C -6.00867500 3.25587700 -0.61973200
C -7.88933400 2.28251100 0.51220200
C -6.56345000 4.51577300 -0.45415800
H -5.05651500 3.15270100 -1.12917900
C -8.44126100 3.54599900 0.65880100
H -8.40653500 1.42228100 0.92213700
C -7.79783400 4.69353000 0.18005900
H -6.03267200 5.37705200 -0.84279900
H -9.38577100 3.64510800 1.18540200
C -6.80774100 -0.33945100 -0.41119400
C -8.04499900 -0.36355000 -1.06412400
C -6.29446900 -1.54735800 0.07532700
C -8.74430100 -1.55316000 -1.20241000
H -8.46385500 0.55221600 -1.46584000
C -6.99739200 -2.73234200 -0.07917800

H -5.33079200 -1.55829400 0.57321900
C -8.24138500 -2.76648100 -0.71836400
H -9.70821400 -1.53653600 -1.70205700
H -6.56783600 -3.65013900 0.30572600
C -8.43973700 6.01869500 0.31402300
C -9.79455800 6.17091200 0.03558400
C -7.73738300 7.17091900 0.73868500
C -10.46585800 7.38629800 0.15158100
H -10.35079000 5.30320500 -0.30560100
C -8.38677200 8.39228600 0.85920600
C -9.74868900 8.50363200 0.56404100
H -11.51934200 7.44260200 -0.08944100
H -7.86498200 9.28125100 1.18960400
C -8.98840100 -4.02473100 -0.93359500
C -9.17331100 -4.98809200 0.08538400
C -9.55879000 -4.29756000 -2.17297000
C -9.89047500 -6.15143300 -0.16069300
C-10.28267900 -5.45738900 -2.44106300
H -9.41792800 -3.57884300 -2.97444100
C -10.44348600 -6.38977200 -1.42224000
H -10.04696800 -6.89450000 0.61072100
H -10.69358000 -5.61697400 -3.42938600
O -6.42852700 7.00600800 1.04058000
O -8.64203000 -4.69935300 1.29637100
O -10.27445800 9.74376900 0.71180500
O -11.11721500 -7.55866200 -1.54871900
C -11.65238300 9.91654300 0.43274100
H -12.27177800 9.30032800 1.09274800
H -11.86643100 10.96851700 0.61554300
H -11.87955100 9.67640600 -0.61114300
C -5.69316400 8.12649100 1.49634500
H -5.66415900 8.91839500 0.74064400
H -6.10983200 8.52442400 2.42763700
H -4.68202900 7.76550400 1.67897900
C -8.82432700 -5.62469900 2.35208300
H -9.88625200 -5.76595600 2.57914900
H -8.36866000 -6.59229200 2.11699700
H -8.32507100 -5.19202800 3.21793700
C -11.70120000 -7.85387700 -2.80497800
H -10.94104000 -7.92210100 -3.59012300
H -12.18866200 -8.82085300 -2.69002700
H -12.44742300 -7.10229200 -3.08294500
N 0.08886400 2.69516400 -0.93997300
S 1.41599400 3.54591400 -1.29191600
N 2.55789600 2.43263000 -1.03264600
C 7.89146800 0.42268000 -0.32071900
C 8.44711200 -0.83196600 -0.51580100
C 6.46675900 0.30605100 -0.31517900
C 7.36767300 -1.88882200 -0.66077500
C 6.12862700 -1.02429500 -0.51095200
C 4.02102700 -0.05465600 -0.36149100
C 10.36376000 0.46519100 -0.33462500
C 4.74560800 -1.22789500 -0.53427100
C 9.84716800 -0.80770200 -0.52374200
S 5.10057900 1.32636000 -0.19512900
S 9.07751200 1.64339100 -0.14554100
C 11.75223100 0.87321700 -0.28300900
C 12.12443800 2.19119900 0.03593100
C 12.78403700 -0.04994500 -0.55043700
C 13.45136600 2.56016700 0.08734700
H 11.36536000 2.93561300 0.25307100
C 14.10986800 0.31758600 -0.49589100
H 12.54041700 -1.07297400 -0.81116900
C 14.48322200 1.63765000 -0.17273300
H 13.70854800 3.58468800 0.33786900
H 14.86176200 -0.43078100 -0.70978000
C 15.83126500 2.12703500 -0.08349900
H 15.90663100 3.17766100 0.18228900
C 17.03651500 1.51636300 -0.26862900
C 17.20613700 0.15072400 -0.62025700
N 17.36296600 -0.95845000 -0.90651300
C 18.30872400 2.25249000 -0.11117100
O 19.39816000 1.74836000 -0.26462800
O 18.13935400 3.53667800 0.21931700
H 19.01523200 3.93769200 0.30121600
H 4.25889500 -2.18072800 -0.67985700
H 10.47206600 -1.68245400 -0.65429900
C 7.46369600 -2.94012600 0.45267900
H 6.64096700 -3.65595200 0.37155300
H 8.40409400 -3.49272500 0.37192000
H 7.41973000 -2.47268900 1.43956900
C 7.41951800 -2.55761600 -2.04093600
H 8.35247500 -3.11625500 -2.15790100
H 6.58682700 -3.25771600 -2.15352900
H 7.35775800 -1.81440500 -2.83987700
C -3.90920800 1.40230400 3.31569100
C -4.60917400 1.21714200 2.15855800
C -3.93916900 0.95073000 0.92876200
C -2.50579900 0.85180800 0.93157500
C -1.81557000 1.06228600 2.16254700
C -2.49201100 1.33255700 3.31648800
C -4.64238500 0.76036300 -0.27153700
C -1.82268400 0.53557400 -0.25398300
C -2.53084600 0.34555700 -1.45150200
C -3.96155400 0.47297800 -1.46549500
C -4.65103200 0.31401800 -2.70291900
H -5.72828500 0.43071100 -2.71769000
C -3.97514500 0.02277400 -3.85249300
C -2.56479900 -0.13190300 -3.83642600
C -1.86790600 0.02688500 -2.67395000

H -4.43676900 1.60301200 4.24239700
H -5.69141800 1.27087300 2.16178400
H -0.73259700 1.00263200 2.16923100
H -1.94723200 1.49017500 4.24152500
H -4.51639200 -0.09122300 -4.78599800
H -2.04082600 -0.37289900 -4.75548100
H -0.78941200 -0.08856000 -2.66755800
C 1.46514100 -3.04070800 1.06642200
C 0.03847500 -2.98962800 0.77525400
N 2.24593300 -2.06482400 0.69434700
N -0.50254000 -1.87941400 0.36636600
C 2.08533900 -4.13966100 1.83898700
C 1.46376200 -4.65850300 2.97905500
C 3.34070400 -4.62840500 1.46764100
C 2.08873300 -5.64527800 3.73088400
H 0.49636500 -4.27879600 3.29039700
C 3.95525800 -5.62815400 2.20934300
H 3.82595000 -4.22463300 0.58632200
C 3.33144600 -6.13832400 3.34452800
H 1.60332600 -6.02972800 4.62178400
H 4.92360600 -6.00940300 1.90199600
H 3.81346900 -6.91609000 3.92770300
C -0.85639300 -4.16207800 0.89122400
C -2.16145800 -3.98645600 1.36106100
C -0.44721200 -5.43147800 0.47203000
C -3.03423700 -5.06389000 1.42928100
H -2.47972500 -2.99881700 1.67659500
C -1.32899500 -6.50365800 0.52268600
H 0.55646600 -5.57996000 0.08813400
C -2.62077500 -6.32508100 1.00861200
H -4.04038100 -4.91925000 1.80938300
H -1.00602100 -7.48135000 0.18077200
H -3.30450600 -7.16631200 1.05679400

HJ105
N 8.80590562 -0.13728401 -0.53312504
C -2.47147518 -1.75787513 -0.74257405
C -2.87152721 -1.04918707 0.36835303
C -3.54915325 -2.11988315 -1.61297212
C -4.21048530 -0.71239505 0.65914205
C -4.91886335 -1.77054513 -1.31626510
C -5.29410336 -1.04412708 -0.13300901
C 8.65081562 1.26918009 -0.58999704
C 9.09319468 2.07281015 0.46359203
C 8.06188960 1.87878314 -1.69862912
C 8.94906664 3.45028025 0.40744803
H 9.55822569 1.61187012 1.32876509
C 7.90888059 3.25710624 -1.74051313
H 7.71535354 1.27010409 -2.52725418
C 8.35205262 4.07498129 -0.69464305
H 9.30249169 4.05206329 1.23645109
H 7.43416955 3.70768327 -2.60657419
C 10.04119672 -0.67447505 -0.09885801
C 11.24524880 -0.13635601 -0.55487004
C 10.08118474 -1.73898113 0.80572706
C 12.45508388 -0.65217704 -0.11294401
H 11.23213483 0.69628805 -1.25022709
C 11.29416081 -2.25730916 1.23097609
H 9.15366963 -2.16446016 1.17438209
C 12.51031692 -1.72744712 0.78136906
H 13.37832398 -0.20702101 -0.47051003
H 11.29745779 -3.08513022 1.93018614
C 8.22660057 5.54510938 -0.79584306
C 8.56310163 6.19605645 -1.97855414
C 7.75591858 6.34648344 0.27016602
C 8.46287361 7.57541553 -2.14373215
H 8.93907662 5.60174642 -2.80567320
C 7.64853253 7.72327554 0.12645501
C 8.00373757 8.34009962 -1.07673308
H 8.75046462 8.02544956 -3.08485122
H 7.28303351 8.35002259 0.92970407
C 13.81629700 -2.29319116 1.18352508
C 14.12794699 -2.62391719 2.52290118
C 14.80751208 -2.50734318 0.23066902
C 15.37243911 -3.14402923 2.85244121
C 16.06177016 -3.02811622 0.53962804
H 14.58557704 -2.27374917 -0.80607206
C 16.33916318 -3.34965624 1.86386013
H 15.62924613 -3.39463824 3.87365428
H 16.78768123 -3.18229823 -0.24790802
O 7.39853052 5.69866339 1.40282310
O 13.16915095 -2.38115517 3.44598925
O 7.86371759 9.68692372 -1.10077108
O 17.51287128 -3.86636928 2.29932616
C 8.20028459 10.36782476 -2.29669216
H 7.57613055 10.03478971 -3.13234423
H 8.01262056 11.42306982 -2.10430015
H 9.25627769 10.22764275 -2.54984718
C 6.89691049 6.45880544 2.48714018
H 7.64069753 7.17669153 2.84804721
H 5.97984045 6.98930553 2.21053716
H 6.67493948 5.74201242 3.27638124
C 13.44903597 -2.66923419 4.80381334
H 13.65460197 -3.73460627 4.95074636
H 14.29573905 -2.07888015 5.16927837
H 12.55233793 -2.39529517 5.35776841
C 18.53475134 -4.08849429 1.34348109
H 18.83477235 -3.15377823 0.85857206
H 19.37982539 -4.49570232 1.89647113
H 18.21743131 -4.80965434 0.58317204

N -3.41239725 -2.75358020 -2.77219720
S -4.89622235 -2.91422421 -3.40963924
N -5.76708140 -2.17300816 -2.25476616
C -10.53552278 -0.28148602 0.31547902
C -10.81711080 0.46648403 1.44776810
C -9.12145465 -0.46777803 0.21973102
C -9.54430569 0.83173006 2.18934516
C -8.51652663 0.16609301 1.29206609
C -6.65994947 -0.69945405 0.19021601
C -12.95695093 0.11518701 0.61885304
C-7.12531752 0.03578800 1.27854809
C -12.18858789 0.69077205 1.61946212
S -8.00092558 -1.22485109 -0.82634806
S -11.94606384 -0.71740905 -0.54870304
C -14.39688905 0.13802101 0.46602303
C -15.03385510 -0.46170803 -0.63481905
C -15.21271109 0.76913605 1.42754310
C -16.40575718 -0.43111703 -0.76324406
H -14.44779903 -0.95759207 -1.40174010
C -16.58315221 0.80149706 1.29857609
H -14.76305706 1.24046409 2.29326516
C -17.22179623 0.19991601 0.19551801
H -16.86895624 -0.90290307 -1.62417512
H -17.16155522 1.29852310 2.06638715
C -18.63961035 0.17266701 -0.03672700
H -18.93272735 -0.34814503 -0.94400507
C -19.68845643 0.68004005 0.67208605
C -19.56845941 1.40279010 1.88890314
N -19.48960639 1.99331114 2.87979720
C -21.08426351 0.51345804 0.21481502
O -22.04212561 0.94633607 0.81459306
O -21.18792753 -0.16557801 -0.93206307
H -22.12682662 -0.22517802 -1.15487408
H-6.46871445 0.45638903 2.02497815
H -12.61661090 1.25390809 2.43927117
C -9.52824267 0.23449902 3.60273326
H -8.57221460 0.44249403 4.09140130
H -10.32382272 0.67536105 4.21009130
H -9.67391368 -0.84826006 3.57318226
C -9.34330469 2.35214117 2.24255916
H -10.13918675 2.81974320 2.82919520
H -8.38643062 2.59183019 2.71486320
H -9.35246967 2.78471320 1.23892109
C 5.68523239 -2.67022419 -1.63221812
C 5.40227338 -1.44923210 -1.03099807
C 6.41963844 -0.59996304 -0.65503605
C 7.75056658 -0.98126907 -0.89930206
C 8.01093259 -2.21708416 -1.51536211
C 6.98732249 -3.07835622 -1.88179613
C 3.42315825 -2.61086919 -1.49153011
C 3.90780428 -1.30281609 -0.89221706
H 6.20848143 0.34823503 -0.17298901
H 9.03736166 -2.50317118 -1.71208012
H 7.22472553 -4.02281229 -2.35667517
C 4.39838332 -4.63813133 -2.52228318
H 3.77859727 -4.59004233 -3.41849725
H 5.39894739 -4.95147335 -2.80619520
H 3.98289128 -5.36718337 -1.82440113
C 2.11243915 -3.11039522 -1.65161412
H 2.03899715 -4.09787929 -2.10290115
C 0.89324106 -2.58449019 -1.34052309
C 0.15021301 -1.41455010 -0.75679305
C -0.42483503 -3.24480223 -1.56820611
C -1.10328408 -2.10059715 -0.99688007
O -0.71805905 -4.34029231 -2.02234915
O 0.47385603 -0.32565302 -0.30685802
C 3.40432825 -0.10405301 -1.71383512
H 3.87262428 0.80718206 -1.33225110
H 3.67141126 -0.21053402 -2.76832420
N 4.47175532 -3.32859824 -1.89519113
F -4.37926432 -0.04665300 1.80527113
F -1.97837114 -0.68646605 1.28695609
H 2.32336717 -0.00189900 -1.61282412
C 3.50053925 -1.18663709 0.58538904
H 3.96952229 -0.29658102 1.01309107
H 2.41944817 -1.07859708 0.67497205
H 3.83521728 -2.05722615 1.15509008

HJ106
N 9.11719700 -0.55886700 -0.25036700
C -2.17249400 -1.76115200 -1.35201700
C -2.62736300 -0.43096200 -1.26131100
C -3.17357700 -2.75228200 -1.21256900
C -4.00603400 -0.08542100 -1.02373600
C -4.55995200 -2.41226300 -0.97208600
C -5.02643200 -1.06722700 -0.87259300
C 8.98906800 0.83054000 -0.01670800
C 9.43601300 1.39206300 1.18238300
C 8.42134100 1.66819000 -0.97835200
C 9.31969400 2.75440000 1.40958300
H 9.88371400 0.75430900 1.93733900
C 8.29545500 3.02847500 -0.73637300
H 8.07039100 1.25101000 -1.91636300
C 8.74598500 3.60530100 0.45665800
H 9.67772800 3.16511600 2.34630500
H 7.83730800 3.65688800 -1.49380500
C 10.32368500 -1.21074500 0.09736700
C 11.55620600 -0.62150600 -0.18800800
C 10.30563100 -2.44899800 0.74540000
C 12.73644000 -1.25641800 0.17140100

H 11.58856700 0.34281600 -0.68397500
C 11.48954000 -3.08234200 1.08869500
H 9.35530800 -2.91814200 0.97790100
C 12.73333500 -2.50165100 0.81027000
H 13.68243300 -0.77125900 -0.04820700
H 11.44782100 -4.04291600 1.58857900
C 8.65419700 5.06773500 0.65693800
C 9.01200200 5.93735700 -0.36856900
C 8.19651700 5.64719800 1.86318800
C 8.94350800 7.32351700 -0.25072200
H 9.37925100 5.51462700 -1.29867900
C 8.12073900 7.02671400 2.00162800
C 8.49576900 7.86637500 0.94873900
H 9.24625300 7.94838200 -1.08068200
H 7.76569100 7.48575200 2.91534200
C 14.00933600 -3.17921300 1.12671500
C 14.25894800 -3.79418200 2.37562500
C 15.03222500 -3.21921300 0.18443700
C 15.47556100 -4.41333200 2.62916600
C 16.25972200 -3.83462600 0.41820100
H 14.85773100 -2.76459100 -0.78589500
C 16.47540300 -4.43794400 1.65246300
H 15.68513100 -4.88205200 3.58206100
H 17.01272800 -3.84243400 -0.35890000
O 7.81996300 4.79186800 2.84139800
O 13.27041300 -3.71855200 3.29627100
O 8.38599100 9.19316300 1.19786500
O 17.61708500 -5.07407400 2.00775500
C 8.74427400 10.09362200 0.16446900
H 8.11735300 9.95060900 -0.72174500
H 8.57942900 11.09235300 0.56590500
H 9.79816700 9.98304900 -0.11119200
C 7.32456600 5.32793800 4.05473700
H 8.07909700 5.94299600 4.55595400
H 6.42013200 5.92196600 3.88701300
H 7.08253100 4.47153300 4.68239400
C 13.48635200 -4.29874000 4.56973300
H 13.65837600 -5.37727500 4.49203200
H 14.33192000 -3.82892700 5.08286500
H 12.57476800 -4.11904100 5.13781100
C 18.66914000 -5.12691400 1.06045200
H 19.01322900 -4.12256300 0.79266200
H 19.48057300 -5.66977000 1.54279900
H 18.36237900 -5.66189300 0.15570800
N -2.95360500 -4.07195000 -1.22545300
S -4.37869000 -4.79264700 -0.98834300
N -5.33201700 -3.49561100 -0.84195800
C -10.23997600 -0.86805500 0.17655900
C -10.69860300 0.43882800 0.09013300
C -8.84273600 -0.89330100 -0.11233600
C -9.57255900 1.38597700 -0.28102900
C -8.42244200 0.40069900 -0.38190500
C -6.41979000 -0.76601800 -0.64244500
C -12.65607400 -0.66879600 0.66490800
C -7.06277500 0.47472000 -0.67717500
C -12.06589400 0.55072100 0.36547700
S -7.58349900 -2.04176400 -0.23888900
S -11.48055800 -1.96952600 0.59683800
C -14.03819700 -0.93917800 0.99901000
C -14.47040200 -2.22254100 1.37860000
C -15.00329700 0.08821500 0.95497200
C -15.79033300 -2.46099200 1.69480700
H -13.76279500 -3.04349100 1.43171400
C -16.32151600 -0.14920300 1.27336400
H -14.71345700 1.08962500 0.65989500
C -16.75486200 -1.43537900 1.65398900
H -16.09461700 -3.46166200 1.98552200
H -17.02110500 0.67483600 1.22189200
C -18.10102600 -1.79397600 2.00524600
H -18.22879500 -2.84037900 2.26786700
C -19.25103400 -1.06353900 2.07079300
C -19.34688400 0.32271100 1.77688400
N -19.44291300 1.45068700 1.54169000
C -20.53571100 -1.67741200 2.46758000
O -21.57769300 -1.06575300 2.53694900
O -20.43581300 -2.98150300 2.74453000
H -21.31501500 -3.29798000 2.99257700
H-6.51894700 1.37685100 -0.90961600
H -12.61588700 1.48354700 0.35689700
C -9.32778300 2.42698700 0.81922700
H -8.46341900 3.04779300 0.56631700
H -10.19822900 3.08090800 0.92443700
H -9.13817800 1.94454200 1.78143500
C -9.84039400 2.07506700 -1.62570800
H -10.71830900 2.72321300 -1.55223200
H -8.98372000 2.69191200 -1.91156800
H -10.01692400 1.34013500 -2.41517500
C 6.00614200 -2.71616500 -1.99470600
C 5.71646300 -1.67375900 -1.12167900
C 6.73064400 -0.95426800 -0.52826300
C 8.06376400 -1.27975500 -0.83084500
C 8.33156000 -2.33169100 -1.72143100
C 7.31111100 -3.06668300 -2.30790100
C 3.73987300 -2.65273900 -1.90827100
C 4.21999200 -1.55033000 -0.97977500
H 6.51504600 -0.14860200 0.16509800
H 9.36095100 -2.57206800 -1.96023100
H 7.55279300 -3.86780100 -2.99605400
C 4.72879000 -4.38887400 -3.36935300
H 4.28130700 -4.06715400 -4.31177800

H	5.73442500	-4.75226300	-3.56172600
H	4.13960800	-5.20061100	-2.94065300
C	2.43281900	-3.05221100	-2.24821300
H	2.36353700	-3.86628500	-2.96697100
C	1.20525800	-2.59367400	-1.85742200
C	0.45859600	-1.60792400	-1.00656000
C	-0.10635700	-3.10064400	-2.34210300
C	-0.79643600	-2.10225500	-1.54956400
O	-0.39494600	-3.98403700	-3.13780800
O	0.78411600	-0.78960700	-0.16102600
C	3.73793600	-0.17148600	-1.45741600
H	2.65659800	-0.08642100	-1.34766200
H	4.20564600	0.60117400	-0.84135000
N	4.79621200	-3.27541100	-2.43852800
H	4.01932800	0.00455900	-2.49884900
C	3.78689100	-1.83007100	0.46933100
H	4.24936400	-1.08861300	1.12644100
H	2.70388300	-1.74401100	0.56398000
H	4.11256400	-2.82270800	0.79077600
C	-3.44152000	2.16016600	-1.14519900
C	-2.05234600	1.80571600	-1.34670200
N	-4.34025000	1.22788300	-0.97980600
N	-1.70432600	0.55153600	-1.42916300
C	-3.94523400	3.55301200	-1.15699500
C	-4.88208600	3.94939600	-0.19794900
C	-3.56101600	4.45946500	-2.14936600
C	-5.41010500	5.23388100	-0.21885600
H	-5.18353700	3.24456000	0.56975800
C	-4.10529000	5.73718000	-2.17948300
H	-2.84861700	4.16088200	-2.91088900
C	-5.02412200	6.13066400	-1.21091600
H	-6.12488400	5.53522400	0.53998200
H	-3.81116900	6.42752900	-2.96316400
H	-5.43966300	7.13283800	-1.23075200
C	-0.95972400	2.80119300	-1.44240300
C	-0.85542800	3.85269400	-0.52765500
C	0.02636100	2.64745500	-2.42015100
C	0.21679500	4.73365500	-0.59254300
H	-1.60394300	3.97346000	0.24819900
C	1.08652400	3.54134100	-2.49579100
H	-0.05012600	1.82437200	-3.12248400
C	1.18523400	4.58602500	-1.58102700
H	0.29624300	5.53736900	0.13199700
H	1.83881000	3.42110600	-3.26854800
H	2.01653800	5.28132300	-1.63603800
